# Acceptance of Insect-Based Food Products in Western Societies: A Systematic Review

**DOI:** 10.3389/fnut.2021.759885

**Published:** 2022-02-21

**Authors:** Tieneke Kröger, Jacqueline Dupont, Lucy Büsing, Florian Fiebelkorn

**Affiliations:** Department of Biology Didactics, Osnabrück University, Osnabrück, Germany

**Keywords:** edible insects, entomophagy, novel food, acceptance, willingness to consume, Western countries

## Abstract

Consuming insects is a possible alternative to meat consumption that has few detrimental impacts on the environment and human health. Whether novel foods made from insects will become established in Western societies in the coming years depends largely on their acceptance by the respective populations. Numerous studies on the acceptance of insects as a novel food have already been conducted. In this systematic review, the main findings of quantitative, experimental, and tasting studies on the acceptance of insects as a novel food are summarized. The present paper is designed to serve as an orientation for practitioners in the food industry and provides information useful for the design of marketing strategies and target group-oriented product development. In addition, we highlight in which fields future studies could be conducted to further improve the understanding of the acceptance of insects as food in Western societies.

## Introduction

The United Nations (UN) predicts that the world population will increase to ~9.7 billion in 2050 (1). With the fast growth in the global population, providing sufficient food supplies, especially dietary protein, is arising as an urgent public health and environmental issue, as meat still constitutes the main source of protein for the majority of Western societies (2). The ethical and environmental concerns associated with conventional meat production will be further aggravated as the millions rising out of poverty in developing countries contribute to a predicted 73% increase in demand for meat by 2050 (3). Alongside changes to industrial animal husbandry, various types of meat substitutes and alternative protein sources may contribute to solving these issues. One proposed solution is entomophagy, the consumption of insects, which are regarded as one of the most sustainable animal protein sources for human consumption (4, 5).

Various legal regulations around the world govern the production and marketing of edible insects (6). To provide a general overview, the legal framework of the European Union is briefly described below. According to Regulation (EU) 2015/2283 of the European Parliament and of the Council, edible insects and their parts are regarded a novel food, since they were “not used for human consumption to a significant degree within the Union before 15 May 1997” [(7), p. 7]. This new Regulation, applicable as of January 1, 2018, facilitates the introduction of innovative foods like edible insects to the EU market, while maintaining a high level of food safety.

In June 2021, the yellow mealworm—the larvae of the beetle *Tenebrio molitor*—became the first insect to be approved as a novel food in the EU (8). Another authorization for the migratory locust (*Locusta migratoria*) as a novel food was issued in November 2021 (9). Furthermore, there is already a scientific opinion for the use of house crickets (*Acheta domesticus*) as a novel food, thus an approval can be expected soon (6, 10). In addition, applications for novel food products from the following insect species have already been submitted: tropical (banded) house cricket (*Gryllodes sigillatus*), lesser mealworm (*Alphitobius diaperinus*), black soldier fly (*Hermetia illucens*), and the honey bee (*Apis mellifera*) (6). Selected insect species can already be sold as food in the EU if they were marketed before 1 January 2018 and an application for authorization as a novel food was submitted by January 2019 at the latest (6). An outstanding environmental benefit of rearing edible insects is based on the high feed conversion efficiency of insects. Due to their being poikilothermic, insects are remarkably efficient at converting feed into protein. House crickets, one of the four most important edible insect species in Western countries (11), need between one-twelfth (cattle) and half (pigs/chicken) as much feed to produce the same amount of protein as traditional livestock (12). Additionally, insects can be reared on organic waste streams and thus help reduce environmental contamination. The large-scale cultivation of cows, pigs, and poultry to meet global demand for animal proteins has severe consequences on natural resources and greenhouse gas emissions. In contrast, insects are reported to emit substantially fewer greenhouse gases and less ammonia than cattle or pigs, and their rearing requires significantly less land than conventional animal husbandry (12–14).

In addition to this environmentally friendly conversion efficiency, insects provide nutritional benefits, including high fat, vitamin, fiber, and mineral content (15–17). For these reasons, the consumption of insects could contribute to solving future food insecurities. However, the nutritional value as well as the sustainability potential of edible insects is highly variable due to the wide range of insect species (12, 15). Detailed nutrient and sustainability analyses are only available for a few of the 2.111 edible insect species currently recorded (16–18).

In addition, in the EU only three detailed scientific opinions on the safety and health aspects of insects as novel foods have been issued, covering yellow mealworms, migratory locusts and house crickets (10, 16, 17). For example, in the case of yellow mealworms, and migratory locusts, the concentration of contaminants depends largely on their presence in the feed. However, according to the European Food Safety Authority's Panel on Nutrition, Novel Foods, and Food Allergens, the two insect species do not cause any safety risk if the feed is produced in accordance with EU guidelines (16, 17). Nevertheless, allergic reactions with yellow mealworms, migratory locusts, and house crickets may occur if they are consumed (10, 16, 17). For instance, although allergens from the feed could be absorbed, such as gluten, proteins from the insects themselves could cause an allergic reaction (10, 16, 17). Nonetheless, Western countries' interest in insects as a potential source of food has grown considerably in recent years, and the environmental and nutritional benefits justify an increasing scientific debate on the topic.

Moreover, the remarkable acceleration of scientific studies in this field of research justifies closer scrutiny. Considering the advancing urgency of providing adequate yet sustainable protein supplies, it is imperative to synthesize the current evidence on the public acceptance of insect consumption. The present systematic review aims to provide a comprehensive picture of the peer-reviewed literature on the acceptance of insect-based foods in Western societies. We hope to equip scholars exploring public acceptance of insects as food with a basis for further research on novel foods and to supply practitioners in the food industry with valuable information on consumers' acceptance of food made from insects.

### What Does This Review Bring That Is New Over Existing Reviews?

This article expands upon the existing review literature by including the latest research and adopting a distinct focus. Hartmann and Siegrist (19) and Mancini et al. (4) conducted systematic reviews with comparable research priorities, as they analyzed European consumers' acceptance of edible insects. Firstly, our work nevertheless contributes to the research on this topic, as it examines the acceptance of consumers from not only Europe, but all Western countries. Secondly, while Hartmann and Siegrist (19) completed their literature acquisition in January 2016, and Mancini et al. (4) in November 2018, 64.4% of papers in our review database were published in 2019 or later. Although Dagevos (20) partly filled this research gap with a literature review of consumer research on edible insects that concentrated on new findings from studies published in 2019, our review closes this gap by adding another 39 articles published in 2020 and the beginning of 2021.

Moreover, we contribute to the existing literature by concentrating on the drivers of and barriers to Westerners' acceptance of edible insects. Multiple systematic reviews have aimed to gain insight into effective ways to promote healthy and sustainable dietary patterns that include the consumption of insects (21–23). However, these studies primarily focused on a few extensively researched determinants that steer consumers to more sustainable and innovative food consumption. In addition, a scoping review by Sogari et al. (24) offers a method-based approach toward investigating consumer perceptions of edible insects. This review, in contrast, provides a comprehensive overview of all drivers of and barriers to the acceptance of insect-based foods that studies have explored thus far, thereby providing more insight into consumer behavior and the effectiveness of interventions.

Additionally, this in-depth analysis was realized with a methodologically advanced approach, applying the Preferred Reporting Items for Systematic Reviews and Meta-Analyses (PRISMA) guidelines and the combined use of the supportive software Sysrev and MAXQDA.

In contrast to the study by Onwezen et al. (25), a recent systematic review that aimed to provide a comprehensive overview of the most relevant drivers of Western consumers' acceptance of five alternative proteins, we concentrated solely on insect-based foods as an alternative protein source. This relatively narrow focus expanded the scope of our paper to a substantially higher number of articles on insects as food. It further facilitated interpretation of our results and implications for possible marketing and education strategies specific to insect-based foods.

Our main contribution to the existing body of literature is the provision of an updated, in-depth overview of factors influencing the acceptance of edible insects. Given the increasing urgency of ensuring a sustainable protein supply, this review could facilitate the understanding of consumer behavior and reveal implications for the promotion of edible insects as an alternative protein source for Western consumers.

## Methods

**Figure 1:**
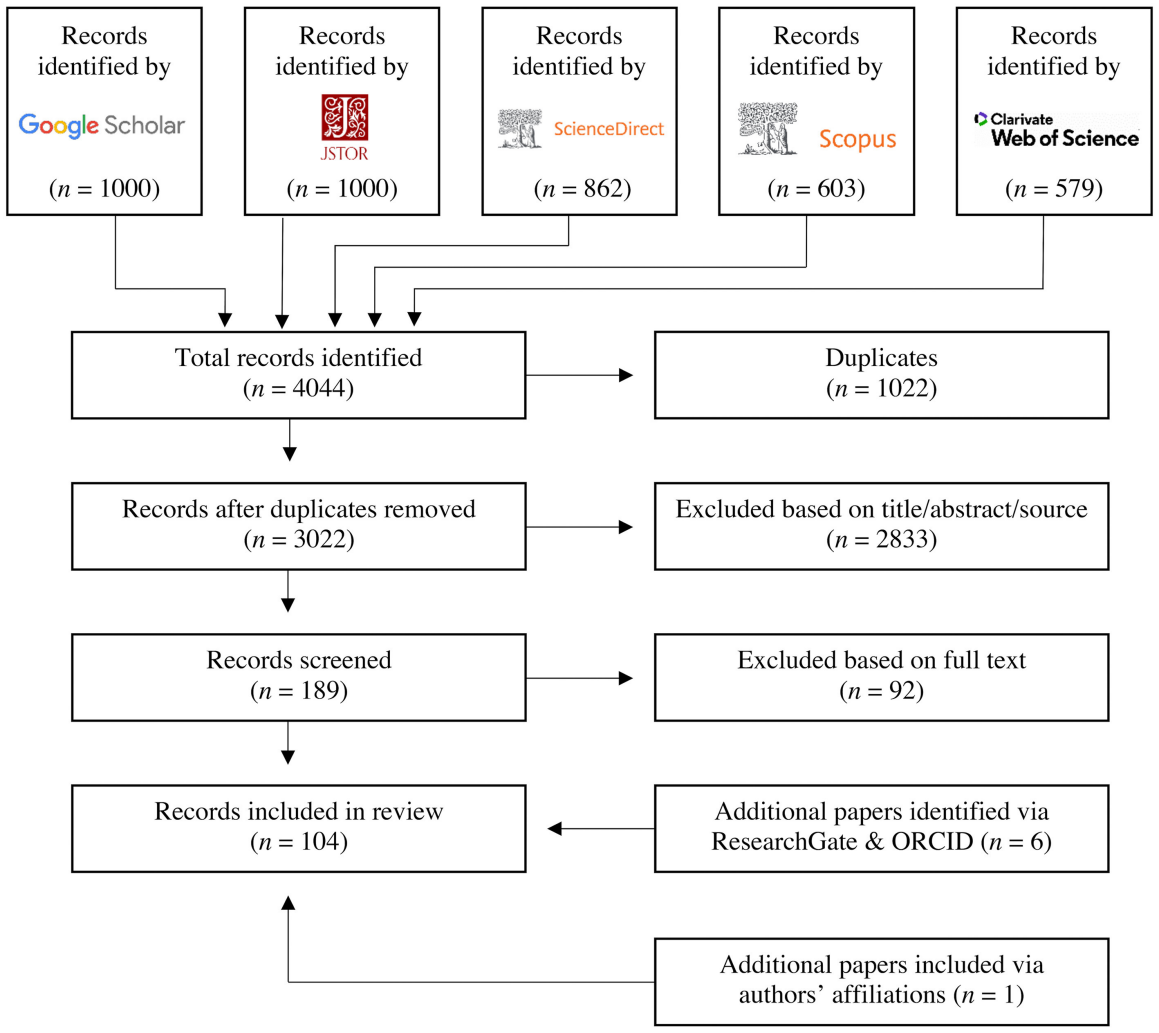
Process for identifying and excluding records based on PRISMA guidelines (26).

This systematic review sought to identify, analyze, and synthesize the findings of empirical studies on consumer acceptance of insect-based food products in Western countries. The review adhered to the PRISMA guidelines for systematic reviews (26). To ensure comparability with other reviews in the identification of relevant scientific literature, we searched the five databases (Figure 1) used by the authors of recent reviews on the acceptance of insect consumption (4, 19, 20).

Thus, Google Scholar, JSTOR, Science Direct, Scopus, and Web of Science were scanned in December 2020 using customized search strings for each search engine. As a result, some studies have already been included in this review that were accepted in 2020 but were not published until 2021. Each search string comprised various alternative terms for “consumer acceptance” and “insects as food,” and wherever technically feasible, a restriction to English language and the publication format article was applied (cf. Supplementary Material). The search strings were tested and refined through multiple rounds. Furthermore, cut-off values were predefined to 1,000 publications per search engine, since hits beyond this threshold were no longer associated with the topic of this review. A total of 4,044 records were identified and populated in Mendeley. After automatic deduplication in Mendeley, the remaining 3,022 articles were imported into Sysrev, an online document review platform. Then we filtered the literature according to the inclusion and exclusion criteria in Table 1.

**Table 1:** Inclusion and exclusion criteria for identifying relevant literature.

**Inclusion criteria**	**Exclusion criteria**
Full-text papers published in a peer-reviewed journal in the English language	Non-peer-reviewed papers
Papers presenting the results of primary empirical studies	Papers that do not present primary research (discussion papers, editorials etc.)
Journals with impact factor (IF)	Journals without impact factor (IF)
Quantitative studies (e.g., taste studies, experimental studies, questionnaire surveys)	Qualitative studies (*post-hoc*)
Focus on willingness to consume insect-based food products	Focus on other aspects of insect-based food products with no focus on consumer behavior
Studies on consumers from Western societies	Studies conducted outside Western societies (traditional societies, Global South societies, etc.)
Studies that have investigated at least one Western society and in which the results for the different societies are presented individually	Studies that have examined both Western and non-Western societies and in which the results are only available as a summary

Qualitative papers were excluded *post-hoc*, as their results were not fit for the systematic structure of our review process. It is hardly possible to quantify the results of qualitative studies—as in the case of regression or correlation analyses—their results could not be directly compared with those of the quantitative studies. Thus, the results of the qualitative studies could not be inserted into the evaluation matrix (cf. Supplementary Table 1). In addition, the results of many qualitative studies are difficult to transfer to the general population, because they often only investigate individual cases or small samples. For these reasons, the results of qualitative studies were not included in the evaluation, though it is well-known that both quantitative and qualitative studies equally contribute to research on the acceptance of insect-based foods. For this reason, and so that the compilation of the identified qualitative studies is not lost, we provide the literature references as Supplementary Material so they can be accessed directly for subsequent studies. Additionally, we included interesting findings from relevant qualitative studies in the discussion section. Research with a mixed methods approach was included in our analysis; however, we only incorporated the quantitative data from these studies (cf. Supplementary Material for bibliography of qualitative and mixed methods studies).

**Figure 2:**
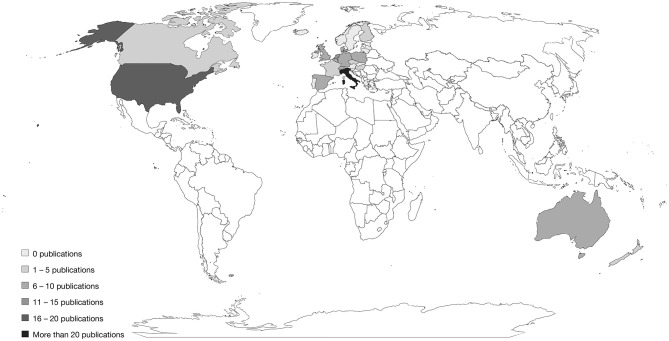
Countries of the Western World and number of publications in the countries. All unmarked countries do not fall under our definition of Western countries (see Section Methods) and were not included in the review.

To accurately define the ambiguous concept of the Western world and provide a politically correct yet wide-ranging working definition for the inclusion criteria, three accepted notions of the West were combined. Firstly, Huntington (27) proposed a theory that the modern world is divided into nine civilizations based on cultural affinities, one of which is the Western civilization. Secondly, Dragolov et al. (28) allocated European Union (EU) and Organization for Economic Co-operation and Development (OECD) member states with a comparable social, political, and economic status to the West. Thirdly, the UN regional group of Western European and other States (29) was considered. Countries mentioned in at least two of these three sources were included in our definition. Hence, in this review, the term “West” covers geographic entities of North America, Australia and New Zealand, Israel, and Europe, with the exception of southeastern European countries. Under this broad definition, 36 countries belong to the Western world (Figure 2).

Based on the inclusion and exclusion criteria, 2,833 papers were excluded in the process of title and abstract screening. Eligibility assessment of the records was performed independently by two authors. Sysrev automatically bundled articles with conflicting labels into a separate folder. These 120 conflicts were resolved after a rescreening process and, if necessary, consultation with all four authors. Then we examined the full texts of the remaining 189 articles and excluded another 92 studies. A total of 97 records were labeled as relevant for the review through this method. Furthermore, we checked the ResearchGate and ORCID profiles of all authors of these 97 studies to find further publications on the same topic. Through these steps, we identified six additional papers. As a last step, we compared our preliminary results with the articles included in the aforementioned reviews on entomophagy (4, 19, 20); however, we did not find any additional studies. Furthermore, one additional paper was included via authors' affiliations. Upon completion of the process, we had gathered 104 papers relevant for inclusion in the review. Articles reporting findings from more than one study were duplicated and analyzed individually, provided that the results were reported separately. Thus, our database comprised 119 studies. However, not all studies are mentioned in the results section. These studies could not show any significant results or were mixed method studies that provided qualitative results (30–35).

In order to collate and synthesize the findings of these studies, we imported the literature into MAXQDA, a software package for computer-assisted data analysis. The publications were analyzed in MAXQDA using codes for bibliographic information and primarily for factors influencing the acceptance of insect-based foodstuff. Even though many different dependent variables were collected in the 119 studies in relation to the action (e.g., willingness to try, willingness to purchase, willingness to consume) and in relation to the insect species and its degree of processing or presentation (e.g., insects in general, specific species, processed/whole insects) these are generally referred to below as the “acceptance” of insect-based food products. Accordingly, the term “acceptance” in this article is understood as a summarizing and superordinate concept and not as an explicit variable. If a study directly surveyed the acceptance, this is indicated below.

Code categories for bibliographic information were set deductively (e.g., title, year of publication, sample), whereas codes for influencing factors were created inductively during the analysis stage (e.g., gender, food neophobia, attitudes). With the help of the quantitative analysis function in MAXQDA, a table with all of these code categories was compiled (cf. Supplementary Table 1). This table shows the factors determining acceptance of insects as food, with subcodes that indicate whether there is a positive, a negative, or no influence on the acceptance of insect-based food products. In the analysis of studies that conducted stepwise regression analyses, we considered the results of the last step only. To create a clear and coherent structure, columns with 0% results (e.g., masculinity) were not removed from the table. Moreover, there are some codes with a dichotomous organization; that is, it only displays whether there is an influence (e.g., amount of substitution, species). In these cases, the nature of this influence is then further explained in the results. To obtain an overview of the most extensively researched factors, we calculated the percentage of the 119 studies exploring each factor. We further calculated the code–subcode ratio to highlight the effects of every factor. It should be noted that these numbers do not necessarily add up to 100%, as several studies reported ambiguous results. In the following, we synthesize the key findings across the literature. The results are presented in clusters of factors related to a common supercategory, such as sociodemographic factors, emotional factors, or social influences.

## Results

Detailed information on the 119 studies included in this review is portrayed in Supplementary Table 1. Due to the substantial size of the file, the table is not integrated into this article, but is available for download as Supplementary Material. Bibliographic data and details on the study procedures are provided and include each study's methods, research question, sample, statistical analysis, insect species and insect-based products investigated, and the dependent variable. This information is followed by the 115 factors identified and examined in the studies. The factors influencing consumers' acceptance of insect-based products are arranged and synthesized into supercategories and are presented and explained in the following subchapters. We recognize that several factors are related to more than one supercategory and will identify those factors accordingly.

### General Findings

**Figure 3:**
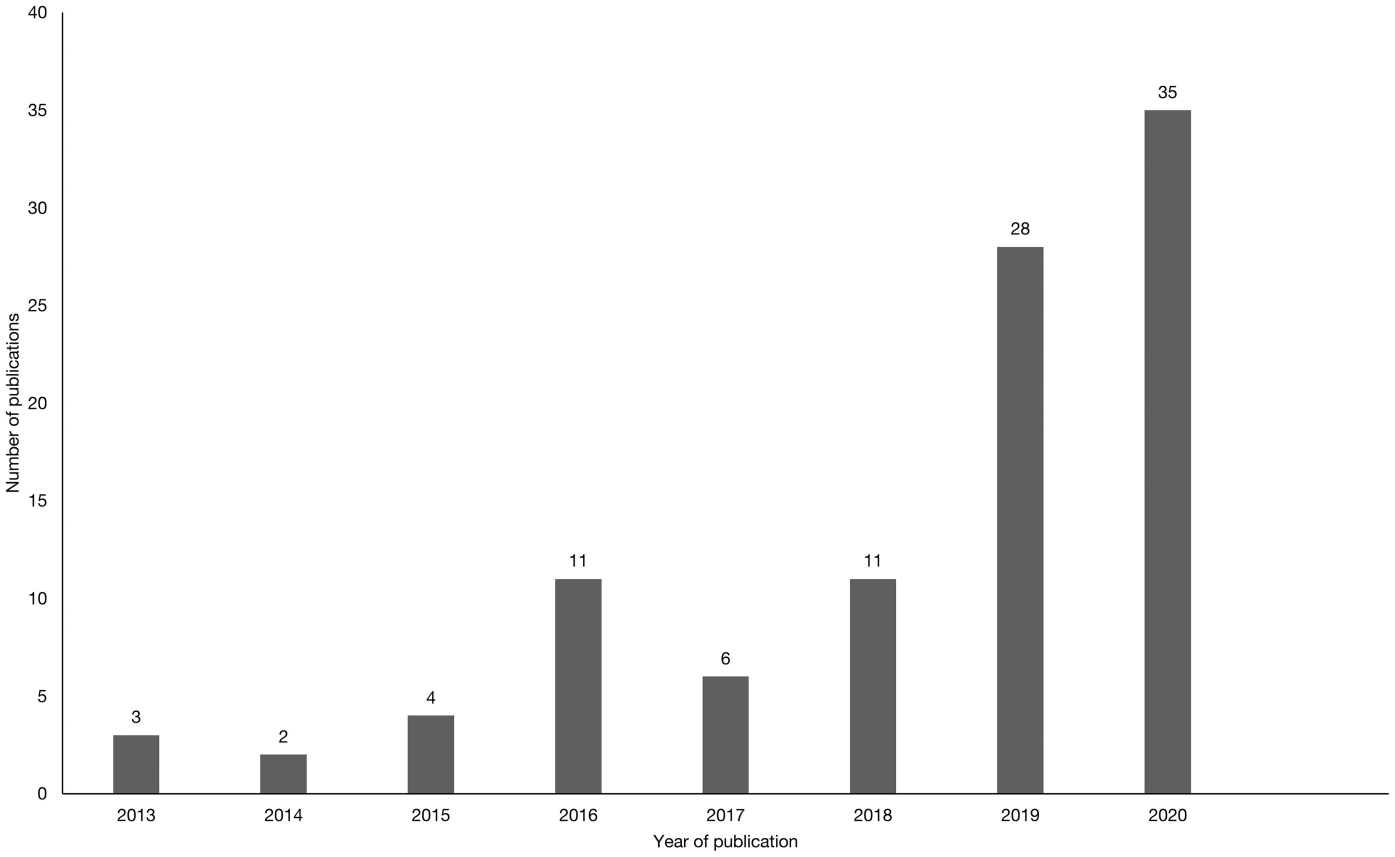
Development of publications on the acceptance of insects as food in Western societies.

The literature search revealed that the research field studying edible insects as an alternative protein source is developing rapidly. The first publications on the topic of edible insects in Western societies date to 2013, whereas approximately two-thirds (64.4%) of the articles in our review were published in 2019 or later (Figure 3). Moreover, results revealed an unequal distribution of articles across countries, with 21 publications exploring Italian consumers' acceptance of insect consumption, 17 concerned with the United States, and 12 with the Netherlands. In several other countries including France, New Zealand, and Canada only one study was conducted (Figure 2).

In addition, during the process of literature acquisition, we identified 18 qualitative studies on the topic (cf. Supplementary Material). A comparison to the 104 articles that included 119 quantitative studies and 11 mixed method approaches highlights an immense overrepresentation of quantitative study designs.

Additionally, we reported the acceptance rate of insects as food in the results table (cf. Supplementary Table 1), which was measured in two-thirds of the studies (2, 36–109). However, the results are only marginally comparable due to the plethora of different methods of measurement and various dependent variables.

### Sociodemographic Factors

The findings related to sociodemographic factors in the reviewed literature on consumer acceptance of insect-based foodstuff vary in their degree of conclusiveness.

#### Gender

The most frequently investigated factor affecting the acceptance of insects as food is gender, a variable known to have an effect on dietary habits (37, 110). Of the 119 studies reviewed, 52 examined gender (2, 36, 37, 39, 41, 42, 44, 49, 51, 55, 56, 58, 61, 62, 64–67, 69, 71–74, 76, 77, 83, 84, 86, 87, 92–94, 96, 97, 99–101, 104–109, 111–117). Of those 52 studies, 71.2% identified masculinity as a positive influence on acceptance of insects as food (2, 36, 41, 44, 51, 55, 58, 61, 62, 65–67, 69, 71, 76, 77, 83, 84, 86, 87, 92–94, 96, 97, 99–101, 104, 105, 112, 114, 116, 117), whereas approximately one-third of those studies (34.6%) found no effect of gender (37, 39, 42, 49, 56, 64, 66, 71, 72, 74, 76, 106–109, 111, 113, 115). Notably, no study found femininity to positively predict the acceptance to eat insect-based foodstuff. Nonetheless, three studies yielded ambiguous results regarding gender (66, 71, 76). The study conducted by Lammers et al. (66), which examined the willingness to consume—including the willingness to try, to buy and to substitute—an insect burger prepared with ground buffalo worms and whole, freeze-dried buffalo worms, suggested that men had a higher willingness to try unprocessed insects. For processed products, however, their data did not indicate any gender difference. The findings of Orsi et al. (76) confirm this observation. While the difference identified by Lammers et al. (66) concerned the preparation of the product, Lundén et al. (71) discovered that the influence of gender was dependent on the insect species: gender was a significant predictor for the acceptance of ants, with males being significantly more willing to try, eat, and cook ants than females, but was not a significant predictor for the acceptance of crickets.

#### Age

Previous reviews have indicated that the concept of eating insects would likely be more appealing to younger people than older people (4, 21, 22). In contrast, the majority of studies (61.1%) in our review concluded that there was no link between age and Westerners' acceptance of edible insects (39, 41, 42, 48, 51, 55, 56, 58, 61, 62, 64, 66, 72–74, 86, 104–106, 108, 109, 117). However, 36.1% of all studies that evaluated the effect of age on consumers' acceptance observed a negative effect of age (2, 64, 65, 67, 71, 76, 77, 87, 92, 93, 97, 99, 115), which means that younger people had a higher acceptance of insect-based foods than older people, while two studies (5.6%) identified a positive connection (49, 107). The percentages add up to more than 100, since Kourimská et al. (64) reported no statistically significant influence of age on the hedonic evaluations of insect products, but on participants' interest in eating insects, with older participants being significantly less interested in insect consumption.

One of the two studies stating a positive connection between age and the acceptance of insect-based food products is the study by Dupont and Fiebelkorn (49), who reported that older participants had a higher acceptance—measured as willingness to consume an insect burger including the willingness to try and to substitute. However, their study is one of the few quantitative studies with a focus on the acceptance of children and adolescents. Hence, the mean age calculated for the sample is 13.67 (*SD* = 2.31). Dupont and Fiebelkorn (49) pointed to a theory that may underpin this exception: according to Nu et al. (118), growing autonomy, increased eating outside the family, and entry into the adult world lead to an expansion of the nutritional spectrum; in addition, Cooke and Wardle (119) provided evidence that adolescents try more food with increasing age. The second study that identified a positive connection between age and acceptance is the study by Zielińska et al. (107), who found older Polish consumers to be more accepting of insects as food than younger individuals.

#### Education

In contrast to previous literature reviews on novel foods (4, 25, 120), who reported higher education to be a driver of the acceptance of insects as food, we obtained ambiguous results regarding the influence of education on the acceptance of insect-based foodstuff. In most studies, “education” refers to the attainment of different school-leaving or vocational qualifications. For example, in a study by Schlup and Brunner (87), education was measured by three different levels: low (no education and compulsory school), middle (apprenticeships and secondary school), and high (technical and vocational training, college, or university). While 42.1%—eight studies (41, 55, 62, 93, 99, 101, 107, 108)—of the 19 studies that examined education identified a higher education level as a predictor of acceptance, 11 studies (57.9%) found that education was not relevant for the acceptance of insects as food (2, 51, 56, 61, 65, 66, 71, 76, 86, 87, 105).

#### Place of Residence and Traditional Food Culture

Results regarding the influence of place of residence on consumers' acceptance of insects as food are similar to the results concerning education, though place of residence was less often examined (42, 65, 67, 71, 87, 93, 99, 105, 116). The place of residence was surveyed with various methods that can be broadly divided into two categories: [1] number of residents and [2] designation of residence (e.g., urban vs. not urban, city vs. rural). If the place of residence had an effect, which was the case in 44.4% of the studies, people living in an urban area or in areas with a high number of residents were found to be more receptive to edible insects (65, 71, 99, 116). In contrast, the majority of studies (55.6%) found that the place of residence had an insignificant effect (42, 67, 87, 93, 105).

Menozzi et al. (73) and Sogari et al. (92) expanded upon place of residence as a predictor and examined the influence of living in a traditional food culture on the acceptance of insects. Both studies were conducted in Italy and attributed stronger attachment to the traditional Mediterranean diet to people from southern Italian regions. Their results revealed considerable reluctance of people from this traditional food culture to consume insects or insect-based products (73, 92).

#### Nationality, Ethnicity, and Travel Experience

Two comparative studies conducted in more than one Western country made comparisons across countries and specifically tested the influence of nationality. Gómez-Luciano et al. (53) compared consumer responses to edible insects in four countries. They found acceptance rates to be considerably higher in the United Kingdom (23.5%) and Spain (~17%) than in the non-Western countries Brazil (6.9%) and the Dominican Republic (~7.5%). In addition, in comparison to residents of Spain and the United Kingdom, people living in the Netherlands and Finland were found to be significantly more willing to eat food from insect-based protein sources, according to Grasso et al. (55). A comparison of the acceptance of insect-based foods at the national level based on the 119 studies analyzed was omitted because the survey methods, insects, products, and contexts differ too much for statements such as “*in a certain country the acceptance is highest/lowest*” to be valid.

Ethnicity does not seem to influence acceptance toward insect-derived foods (106). Woolf et al. (106) differentiated between six groups to elicit ethnicity: Caucasians, Latino Americans, Pacific Islanders, African Americans, Indian Americans, and participants with “*two or more ethnicities.”*

There is some evidence of travel experiences affecting the way Polish consumers relate to entomophagy (116), but this is based on only one study; thus, this finding is not generalizable. Nonetheless, this study found that traveling to North and South America or Asia seemed to have a significant positive effect on the acceptance of edible insects in the diet, whereas a journey to Africa or Europe had no effect (116).

#### Religion

The effect of religious beliefs on the acceptance of insect-based products was measured by Ruby and Rozin (83) in a study of U.S. citizens. “Religious beliefs” referred to the violation of religious principles due to the consumption of insects, assessed with items such as “*It is against my religion to eat insects*.” or “*Spiritual leaders would not approve me eating insects*.” (83). These researchers could not show any influence of religious beliefs on the acceptance of eating insects for U.S. citizens (83). In accordance, Castro and Chambers (38), in a global comparative study of residents of the United Kingdom, the United States, Spain, and Australia, reported that religion had an insignificant effect on the acceptance of insect products. Religion was assessed in the Castro and Chambers (38) study using the item “*Religion does not allow all or certain insects [as food]*.” which was required to be rated on a seven-point Likert scale by respondents. In both studies non-Western countries were also examined (38, 83). Although it is outside the scope of this review, both studies demonstrated a negative impact of religious beliefs for participants from India. For instance, Ruby and Rozin (83) explained the influence of religious beliefs in India (74% Hindu, 10% Catholic, 10% Muslim, and 6% other) by the fact that many Hindus are vegetarians for religious reasons, and insects may be among the animals prohibited as food. For the other countries, Castro and Chambers (38) were unable to reveal any influence of religious beliefs on the acceptance of eating insects.

#### Income and Occupation

The sociodemographic predictors income (67, 87, 93, 105) and occupation (105) (response options: “*student”* or “*other”*) seem to have no impact on people's acceptance of insects as food. Income was assessed in three different ways: [1] monthly income (67, 87), [2] annual income (105) with quantified amounts of money or [3] *via* an assessment of quality of life and the possibility of saving money with items such as “*[I live] well but can only set little money aside*.” (93).

### Personality Factors

This supercategory is comprised of several factors associated with consumers' personalities. In addition to established personality traits, there are a number of more complex constructs in this supercategory. Their allocation to this group will be explained accordingly.

#### Big Five

Thus far, the big five personality traits have only been explicitly tested in an article published by Russell and Knott (85), who examined the influence of agreeableness, the tendency to act selflessly (121), neuroticism, conscientiousness, extraversion, and openness of United Kingdom citizens on their willingness to consume, pay for, and substitute meat with insect products. Their findings suggest that the rate of acceptance of insects as food is significantly higher among extraverted people and those who are open to experience, whereas agreeableness seems to be insignificant. Conscientiousness turned out to be a barrier to acceptance. Neuroticism is the tendency toward negative affect such as anger, anxiety, self-consciousness, irritability, emotional instability and depression (122). There appears to be need for further research on the impact of neuroticism on the acceptance of insects as food as Russell and Knott (85) yielded ambiguous results, with one study reporting no influence of neuroticism and their other study reporting a negative influence.

#### Sensation Seeking

A personality trait closely related to openness and extraversion is sensation seeking (123), which is defined as “*the seeking of varied, novel, complex, and intense sensations and experiences, and the willingness to take physical, social, legal, and financial risks for the sake of such experience*” [(124), p. 27]. The construct was measured in two studies, with both Lammers et al. (66) and Ruby et al. (84) reporting that sensation seeking was a positive predictor of acceptance to insect-based foods.

#### Storytelling

A characteristic loosely connected to extraversion was analyzed in a study by Ruby et al. (84). This variable, called storytelling, describes people's tendency to tell others about their unusual eating experience. Based on their findings, they described potential insect consumers as inclined to be storytellers (84).

#### Mindfulness

An additional marginally examined variable is mindfulness. Chan (125) conducted experiments with United States and Australian undergraduate students to explore the influence of mindfulness on the willingness to try an insect-based drink. Mindfulness “*refers to the state of being aware, taking note of what is going on within oneself and outside of the world*” [(125), p. 375]. Mindfulness proved to be an inhibitor of students' willingness to try an insect-based drink, provided that their disgust sensitivity was comparatively low; otherwise, mindfulness was insignificant. Furthermore, the second experiment conducted by Chan illustrated that mindfulness only indirectly influenced the acceptance of insects as food and was mediated by disgust (125).

#### Attitudes

In comparison to other personality factors, the impact of attitudes has been investigated frequently: 17 of the 119 studies in this review inspected attitudes toward eating insects (37, 49, 50, 62, 70, 73, 75, 99, 100, 104, 107, 115, 126, 127). However, attitudes form an elusive construct (128) and are thus difficult to allocate to a supercategory. Attitudes are defined as a relatively enduring pattern of evaluative responses toward an object, person, group, issue, or concept, ranging from negative to positive. This evaluation of a stimulus object generates affective, cognitive, or conative responses (128–130). Since two of these three attitude dimensions (cognitive and conative) are associated with factors in this supercategory and only the affective response is connected to the emotional factors of the next chapter, we decided to assign attitudes to the personal factors. The results obtained by the studies investigating attitudes are quite conclusive: 82.4% of the studies reported that attitudes are positively correlated with the acceptance of insects as food (37, 49, 50, 70, 73, 75, 99, 100, 104, 107, 115, 126, 127), while three studies (17.6%) found no connection (50, 75). Kornher et al. (62) revealed a negative influence of attitudes on the acceptance of insect-based foods. However, in their study (62) attitudes were coded negatively as they were defined as an aversion to eating insects (i.e., insects are not edible and primitive). Of all of the studies investigating attitudes, only Fischer and Steenbekkers (50) even partially distinguished between the three dimensions of attitudes. Their survey indicates that affective but not cognitive and overall attitudes influence the acceptance of eating insects.

Interest in entomophagy [i.e., general interest in eating insects (131)] is understood as a specific dimension of attitudes, which has been examined in two papers. The three studies (103, 131) found a positive influence of interest in entomophagy on the acceptance to insects.

The influence of another dimension of attitudes, involving a general interest in food health [i.e., consideration of health when eating food (132)] was examined in nine studies (2, 51, 53, 61, 76, 77, 87, 108, 115). Data from four studies (53, 76, 77, 108) identified an increased acceptance of insect consumption in people generally interested in health aspects of their food. However, opposing results were found in five studies reporting that an interest in food health had no impact on the acceptance of insect-based foods (2, 51, 61, 87, 115).

#### Sustainability Consciousness/Awareness and New Ecological Paradigm

Sustainability consciousness is a complex construct that encompasses the psychological components of knowledge, attitude, and behavior and their interaction with environmental, economic, and social aspects of sustainable development (133, 134). The only paper in our review database that explicitly applied the original sustainability consciousness scale by Berglund and Gericke (135) is the study by Lammers et al. (66). However, Lammers et al. (66) only used the subscale for measuring attitudes toward sustainable development in their study. They reported that even though the attitudes toward sustainable development of their German sample were very positive, it did not significantly predict the participants' willingness to consume insects.

Furthermore, twelve studies dealt with sustainability awareness of Western consumers and its effects on their acceptance of edible insects (36, 53, 67, 74, 76, 77, 106). Sustainability awareness describes whether a person considers sustainability in an action or when choosing an object, for instance, when buying food. In a study by Orsi et al. (76), for example, the focus was on sustainable food shopping in general, including aspects such as production methods and packaging. In contrast, a study by Woolf et al. (106) regarded whether insects are a sustainable alternative to conventional meat. The influence of sustainability awareness seems to vary across the literature: three studies (53, 77, 106) reported that it positively influences the acceptance of eating insects, whereas five studies reported no influence (36, 67, 74, 76).

A variable connected with sustainability consciousness and awareness is the New Ecological Paradigm (NEP), a measure of advocacy of a pro-ecological perspective on the world (136). According to the findings of Lombardi et al. (115), NEP scores do not significantly influence consumers' willingness to pay for insect-based food products, except in the case of chocolate bars with mealworm flour as an ingredient. For this product, a positive influence on the willingness to pay could be proven (115).

#### Perceived Behavioral Control and Behavioral Intention

According to Ajzen (137) and “his” theory of planned behavior, perceived behavioral control (PBC), attitudes, and subjective norms are the three decisive factors that determine our intention to display a certain behavior.

PBC describes the perceived ease or complications of a person to execute a certain behavior (137). Behavioral intention, in turn, is said to be the strongest predictor of actual behavior (137). The overarching theory of planned behavior was applied in six studies (72, 73, 75, 99, 101, 126). With the exception of Vartiainen et al. (99), all of the studies found a positive effect of PBC on the acceptance of insects as food (72, 73, 75, 101, 126). In these studies, PBC was operationalized as having the control over consuming insect-based products (73) or including them in one's daily diet (101). Notably, however, the study by Vartiainen et al. (99) measured consumers' avoidance of edible insects as the behavior of interest, which implies that all six studies identified a positive impact of PBC and the acceptance to insect-based products.

Only two of the six studies applying the theory of planned behavior also examined consumers' intention to try insects as an independent variable (72, 73). According to the theory, behavioral intentions include motivational factors and efforts an individual is willing (or unwilling) to invest in to perform a certain behavior (137). Both Mancini et al. (72) and Menozzi et al. (73) yielded results according to expectations of the theory of planned behavior. Since this theory assumes that a behavior is solely predicted by the intention to perform the behavior (138, 139), the two studies theoretically confirmed that the intention to eat insects is a positive predictor for actually consuming insects.

#### Purchase Activism and Trust

According to two studies by Legendre and Baker (140) and Legendre et al. (141), it is important to also investigate purchase activism, a trait describing customers' heightened motivation to express their opinions and make an impact on the marketplace through their purchases (142). Purchase activism seems to positively predict consumers' intention to buy insect-based food, although this was only measured in these two studies. Legendre and Baker (140) and Legendre et al. (141) also pioneered the examination of the effect of trust on purchase intention. On the one hand, trust in the media was examined (141), on the other hand, trust in food regulators was surveyed (140). Again, these two studies detected a connection, with trust indirectly increasing the intention to purchase edible insects via increased purchase activism.

#### Familiarity

Familiarity or knowledge of the concept of edible insects was investigated in 16 studies (2, 37, 49, 51, 61, 66, 67, 76, 86, 95, 100, 104–106, 112, 141). In most of these studies, familiarity refers to whether people have heard of the concept of edible insects. However, other methods of data collection were also chosen, for example Woolf et al. surveyed people's self-reported knowledge (106), while Tan et al. (95) surveyed familiarity with the taste of insect-based foods. In addition, scales with more than one item were occasionally used to assess familiarity; for example Schlup and Brunner as well as Woolf et al. (87, 105). In a study by Woolf et al. (105), both familiarity (on a scale from “*have not heard*” to “*very educated*” about the concept of consuming insect-based products) and knowledge (number of known benefits of entomophagy) were surveyed. Previous reviews on entomophagy have identified familiarity as one of the key drivers of people's acceptance of insects as food (4, 19, 23, 25). Our findings confirm these observations, as 81.3% of the articles measuring the impact of being familiar with the concept of entomophagy observed that it had a positive influence on the acceptance of insects as food (2, 37, 49, 51, 61, 67, 95, 100, 104–106, 112, 141). The results of three of the 17 studies measuring familiarity indicated no significant connection between familiarity and the acceptance of edible insects (66, 76, 86).

Several intervention studies revealed that tasting insect-based food products increased familiarity with and acceptance of insect consumption (4, 70, 113, 143). Alternatively, familiarity can be increased by providing information on entomophagy (43, 67, 112, 144). Knowledge transfer thus proves important in gaining public acceptance of insects as food, as it can shift the perception from insects as pests to insects as a sustainable resource and a critical component of our ecosystem (78).

#### Food Neophilia

Baker et al. (145) defines food neophilia as the “*general human inclination of enjoying a wide range of new and unfamiliar foods*” [p. 96]. As might be expected, both Palmieri et al. (77) and Videbæk and Grunert (103) revealed a positive association between food neophilia and the acceptance of insect-based foods. Notably, however, the study by Videbæk and Grunert (103) only observed this association in the consumer group of potential entomophagists.

#### Neophobia

Neophobia can be defined as a “*persistent and irrational fear of change or of anything new, unfamiliar, or strange*” or to put it more simply as the “*avoidance of new stimuli (especially foods)*” (146). As neophobia by definition is linked closely to the emotion of fear, it should be noted at this point that assigning neophobia to just one supercategory is challenging. Thus, the constructs presented here could also be assigned to the supercategory of emotional factors. However, Pliner and Hobden (147), for example, recommend conceptualizing food neophobia as a personality trait. In addition, several other studies also define food neophobia as a personality trait e.g., (67, 148). Based on this, neophobia and closely related constructs were assigned to this supercategory, knowing that there are close relationships with the emotional factors.

The influence of various sub types of neophobia has been studied in the context of entomophagy. Modlinska et al. (149) investigated general neophobia and concluded that it is a barrier to the acceptance of insect-based food products.

According to a recent review on alternative protein sources (25), food neophobia—the aversion to trying novel foods (147)—demonstrate consistent results in explaining intentions to consume alternative proteins. The findings of this review confirm this observation. Apart from gender, food neophobia is the variable most frequently explored in the articles in our review database. Of the 119 studies examined, 37 investigated the influence of food neophobia (2, 40, 42, 49–51, 56, 59, 61, 62, 66, 67, 71, 72, 74, 76, 84–87, 89, 92, 94–96, 99, 103, 104, 108, 115–117, 127, 131, 143) and 89.2% of these reported a negative influence of food neophobia on the acceptance to insect-based foods (2, 40, 42, 49, 51, 56, 59, 61, 62, 66, 67, 71, 72, 74, 76, 84–87, 89, 92, 94–96, 99, 104, 108, 115–117, 127, 143). However, five studies observed no influence (50, 84, 103, 131). Furthermore, Ruby et al. (84) seemed to discover a product-specific influence of food neophobia, stating that it significantly decreases the acceptance of whole insects, but not insect flour preparations.

A considerably less investigated variant of neophobia is food technology neophobia, the fear of novel food technologies (66). The results obtained are equally informative, however. All four studies that investigated food technology neophobia concluded that it is a barrier to the acceptance of insects as food (2, 66, 77, 87). Additionally, an increased negative influence of food technology neophobia on the acceptance of edible insects in people without prior insect consumption was detected (87).

### Emotional Factors

This supercategory centers around emotional influences on consumer acceptance of insects as food. There are several psychological barriers to the acceptance of eating insects, and the idea of entomophagy can give rise to a variety of emotions.

#### Emotions

Most studies have focused on emotions associated with fear or disgust, and only two papers (52, 75) investigated the influence of emotions in general. Onwezen et al. (75) conducted three studies that examined the effect of both positive (i.e., feeling happy, satisfied or proud when thinking about eating insects) and negative emotions (i.e., feeling angry, sad, or guilty when thinking about eating insects). Interestingly, only positive emotions proved to affect consumers' acceptance; the effect of negative emotions seemed to be insignificant. Gmuer et al. (52) confirmed this finding, as they also pointed to the higher prevalence and intensity of positive food-related emotional states (e.g., well, happy, good).

In addition, Onwezen et al. (75) examined whether ambivalence could predict the acceptance of insects as food. Ambivalence is understood as the simultaneous presence of contradictory attitudes and feelings toward the same person, object, event, or situation (150). Since Onwezen et al. (75) defined ambivalence as an affective variable, we assigned this factor to the emotional supercategory instead of the personality factors, which discussed attitudes. In addition, ambivalence was assessed at product level in the study by Onwezen et al. (75). All three studies in the paper by Onwezen et al. (75) demonstrated a positive influence of ambivalence on the intention to consume an insect-based burger (i.e., consumers with mixed or contradictory feelings, or a feeling of uneasiness when buying an insect-based burger, are significantly more likely to consume this burger). In addition, a positive influence was also found for fresh, dried and fried insects (75).

#### Disgust

The idea of consuming insects commonly triggers adverse emotional responses in Western consumers. One of these prominent negative emotional experiences is disgust. Disgust is an emotional response to an offensive object that arouses a feeling of revulsion (151). Disgust toward unfamiliar food can be explained as a physiological response evolved to protect organisms from ingesting toxins and pathogens (152). Quantitative studies on entomophagy have extensively researched the influence of disgust on Westerners' acceptance of insects as food. Approximately one-third of all studies (30.3%) have explored some form of disgust as a factor triggering rejection of insect consumption (49, 50, 52, 57, 59, 62, 66, 72, 73, 75, 76, 84, 85, 91, 96, 103, 125, 127, 131, 143, 153). The results are described below, subdivided according to the different forms of disgust that were investigated, namely general disgust, food disgust, disgust with insects, and disgust toward eating insects.

According to nine of the ten studies examining general disgust, the emotion of disgust reduces consumer acceptance of insects as food (59, 84, 85, 91, 96, 103, 125, 131). In most studies, general disgust was elicited by stimuli from, for example, the domains of death, bodily precipitation, hygiene, and foulness (154, 155). In a study by Ruby et al. (84) only the sub-dimension core disgust was used for measurement. Russell and Knott (85) revealed an insignificant effect of disgust. However, in a narrower sense, Russell and Knott (85) did not survey general disgust, but rather disgust propensity [i.e., “*the tendency to react with the emotion of disgust*” (156), p. 1249] and disgust sensitivity [i.e., “*the tendency to experience disgust as something horrid*” (156), p. 1249]. The percentages add up to more than 100 (cf. Supplementary Table 1) because Videbæk and Grunert (103) formed participant clusters and found that general disgust influenced only the group of insect opponents, but not the group of insect feeders (i.e., those who feed insects to their animals) or the group of potential entomophagists.

It is informative to investigate not only general disgust, but also food disgust (66), defined as an individual's emotional disposition to react with disgust to certain food-related stimuli (157). According to Hartmann and Siegrist (157) the food disgust scale measures disgust as a character trait, that is, it measures a “*person's emotional predisposition to be more or less easily disgusted by certain food-related cues*” (p. 40). Food-related elicitors of disgust may be for example associated with animal flesh, poor hygiene, human contamination, mold, and decaying vegetables and fruits (157). Food disgust was measured in five studies and yielded clear results (49, 66, 85, 143). With the exception of Dupont and Fiebelkorn (49), every study reported a negative influence of food disgust on the acceptance of edible insects. In the study by Dupont and Fiebelkorn (49), food disgust had a significant negative effect when combined with sociodemographic variables in the second step of their hierarchical regression and only became insignificant in the third step of their analysis, in which they included familiarity, attitudes and food neophobia in the regression model. Furthermore, they referred to a theory suggesting that food disgust sensitivity rises with increasing age (158), which may also mean that children and adolescents experienced less food disgust than adults. Thus, the low average age of their respondents (13.67 years) might explain why their results differ from those of other studies. Disgust with insects has been assessed via a single item in each of two studies, namely “*Insects are disgusting*” (62) and “*[I am] disgusted when seeing insects around*” (73). Both studies were able to consistently show a negative influence of disgust toward insects on the acceptance of food made from insects (62, 73).

Similarly conclusive are the results obtained from the 19 studies (50, 52, 57, 72, 75, 76, 79, 83–85, 103, 125, 127, 131, 153) that specifically examined feelings of disgust toward eating insects: 17 of the studies confirmed a negative influence of disgust toward eating insects on the acceptance of insects as food (50, 52, 57, 72, 75, 76, 83–85, 103, 125, 127, 131, 153). Compared to the first study in the article of Onwezen et al. (75), the remaining two studies revealed insignificant effects of disgust. However, it should be noted that in these two studies, additional factors (e.g., FCMs) were included in the regression model. Disgust with eating insects was sometimes elicited by a single item [e.g., “*Eating insects is disgusting*.” (84)] or by a scale [e.g., the disgust subscale of the Entomophagy Attitude Questionnaire (131)]. In contrast, Poortvliet et al. (79) and Onwezen et al. (75) examined disgust toward eating insects in relation to different products. Participants were shown pictures of various foods made from insects, such as a burger patty, which they were then asked to rate.

### Social Influences

What people perceive to be edible not only changes over time but is also discussed and defined within sociocultural spheres (159). The significance of social and cultural norms in the acceptance of insects as alternative protein source has been acknowledged in numerous recent studies.

#### Subjective Norms

Subjective norms are the felt social pressure resulting from one's perception of the degree to which important others want one to perform a certain behavior (160). Ten studies examined the influence of subjective norms on the acceptance of insects as food (58, 59, 73, 75, 99, 126), eight of which demonstrated their positive influence on the acceptance of insect consumption (58, 59, 75, 99, 126). The remaining two surveys reported the effect of subjective norms to be negligible (73, 75).

#### Source of Social Influence

Collectively, the reviewed literature suggests a link between social influences and the acceptance of insects as food. Nevertheless, research has shown that the source of social influence determines its impact on the acceptance of insect-based foodstuff: while mere exposure to information about someone else consuming insects did not impact one's own willingness to consume—including willingness to consume, to pay and to substitute—insect products (85), recommendations by experts and the experiences of peers significantly increased the acceptance of insects as food (91).

#### Trend and Perceived Normality

Despite the depicted social influence, two studies concluded that the existence of a food trend was not a convincing reason to include insects in one's diet (62, 96). In a study by Kornher et al. (62) the variable is understood as “*preference for street food festivals, food blogs, and the attendance of cooking classes*” (p. 5), which is thus intended to account for respondents' preference for alternative consumption habits. In comparison, Tuccillo et al. (96) asked their participants more generally if a trend would make them to include insects or insects-based-products in their diet. The findings by Russell and Knott (85) expanded upon the effect of social influences. According to Russell and Knott (85), the acceptance of insects was increased when the consumption of insects was perceived to be normal, natural, and common.

#### Social and Financial Acceptability

According to Schäufele et al. (86), feelings of disgust in Western society toward the consumption of insects contribute to the common prejudice that insects are consumed in the developing world because of hunger and that this is just a survival mechanism. In their study, they tested whether their participants had this misconception of entomophagy and to what extent it affected their willingness to try insects as food. Their data on the social acceptability of entomophagy suggests that a more enlightened, positive perception of entomophagy fosters Westerner's willingness to try insects as food (86).

However, financial acceptability—measured by assessing whether a participant believed that insects are a product for people with low financial resources—had no effect on their participants' willingness to try insects as food (86).

### Diet

The studies in our database consistently noted the relevance of dietary patterns to consumers' willingness to incorporate insects into their diet.

#### Dietary Preferences

In addition to examining general dietary preferences, some studies specifically surveyed dietary preferences concerning the consumption of meat. All results of studies that surveyed meat consumption on a general level (i.e., vegetarians and vegans vs. meat eaters) are presented in this section. All studies that have broken down meat consumption more precisely, i.e., studies that, for example, have considered meat consumption per week, or have investigated to what extent the intention to reduce one's meat consumption or meat liking influences the acceptance of insect-based foods, are presented in the following section on “Meat Consumption and Liking.”

Among the studies investigating whether certain dietary preferences promote the acceptance of insects as food (41, 42, 55, 58, 61, 74, 76, 86, 87, 97, 101, 104, 126, 149), data from four studies (55, 74, 86, 87) indicated that diet in general has no influence on the acceptance of insect-based foodstuff. Only one study has shown that general dietary habits have a negative impact on the acceptance of food from insects (97). The five studies examined general dietary behavior, with Grasso et al. (55) differentiating between individuals following and not following a meat limiting diet. Naranjo-Guevara et al. (74) distinguished between four dietary types in terms of animal product consumption: [1] no restrictions, [2] flexitarian, [3] vegetarian, and [4] vegan. Schäufele et al. (86) differentiated between meat eaters and participants who do not eat meat (i.e., vegetarians and vegans). Schlup and Brunner (87) surveyed meat consumption regarding to the month and the week. In contrast, Van Thielen et al. (97) distinguished only between participants who ate meat daily and those with other consumption frequencies. Other studies focused on examining specific dietary preferences affecting consumers' acceptance of insect-based food and indicated which specific aspects of dietary patterns had a beneficial or negative influence (58, 61, 76, 104, 126).

Based on our review of the literature, it is safe to say that a vegetarian diet does not promote the acceptance of eating insects. The literature is discordant, however, as to whether it negatively affects consumers' acceptance to insect-based products, since three studies found a significant negative influence (61, 76, 104), while two studies found no connection (58, 126). Veganism, on the other hand, proved to be a distinct barrier to the acceptance of edible insects (61, 126), although this was rarely investigated. According to the findings of Kane and Dermiki (61), participants who followed a vegan diet rigorously excluded the option to try edible insects. Similarly, Elorinne et al. (126) formed participant clusters and found a significant majority of vegans in the group of consumers unlikely to eat insects.

Further positive influences on the acceptance of insects as food that are associated with dietary patterns are visits to ethnic restaurants (41), familiarity with unusual foods such as offal or raw fish (42), variety-seeking tendency in regard to food choices (149), and being a rational food consumer—a person with interested and critical purchasing behavior (101).

#### Meat Consumption and Liking

All three studies that examined meat consumption as an influencing factor queried weekly meat consumption (49, 62, 66). Two of the studies were unable to demonstrate an association with edible insect acceptance (49, 66), while the third study was able to demonstrate that regular meat consumption was a barrier to edible insect acceptance (62).

When comparing results of consumers' intentions to reduce their meat consumption—surveyed in all four studies via a yes-no question (2, 49, 51, 66)—it seems necessary to consider the studies' dependent variable. Both studies examining the willingness to substitute meat with insect-based products identified intended meat reduction as a positive predictor (2, 51). Dupont and Fiebelkorn (49) and Lammers et al. (66), on the other hand, worked with the “*willingness to consume*” insect-based foodstuff, which measured the willingness to try, to buy [only Lammers et al. (66)] and to substitute as an aggregated variable. Their results conclude that the intention to reduce one's meat consumption has no effect on the willingness to consume insects.

Meat liking was examined in three studies (all using different survey methods), which yielded different results (2, 43, 87). While Schlup and Brunner (87) found no connection between meat liking and the willingness to consume insects, Verbeke (2)—using the dependent variable willingness to substitute meat with insect-based products—found that the surveyed participants who liked meat more were less likely to accept insects as a substitute for meat. In a study by Schlup and Brunner (87), preference for meat was elicited by a single item (“*I especially like meat*.”), whereas Verbeke (2) surveyed the importance of taste in evaluating meat quality. In contrast, in the study by Circus and Robison (43), the willingness to try insect products was reported to be significantly higher for people who liked meat. Circus and Robison (43) surveyed meat liking using a scale on meat attachment that addressed, for example, enjoyment in meat consumption or meat as a part of one's identity.

According to Gere et al. (51) and Kane and Dermiki (61) considering meat to be a healthy dietary protein does not influence the acceptance of edible insects. Data from Schlup and Brunner (87), Brunner and Nuttavuthisit (108), and Verbeke (2), on the other hand, detected conviction about meat as a healthy protein source as a barrier to the acceptance of insect-based food products.

#### Previous Insect Consumption

One of the most obvious and promising influences on the acceptance of insects as food in Western societies is previous insect consumption. Twenty-eight studies in our review inquired about their participants' experience with edible insects, and all of these studies, without exception, confirmed a positive impact of previous insect consumption on consumer acceptance of edible insects (36, 37, 50, 59, 61, 64, 66, 70, 73, 77, 78, 86, 87, 92, 94, 99, 104–106, 108, 109, 112, 113, 117, 143, 144).

#### Seafood and Sushi Consumption

Early adopters of entomophagy are likely to regularly consume fish, seafood, and sushi (108). Moreover, frequency of sushi intake positively influences the participants' acceptance toward edible insects (83, 108).

#### Green Dietary Behavior

This construct refers to the tendency to take environmental impacts into account when making one's food decisions or consuming food products. According to 66.7% of the studies focusing on this variable, a greener purchasing and eating behavior leads to higher acceptance of insect-based foods (2, 55, 62, 78). In contrast, 33.3% of the studies could not show any impact of the green dietary behavior on the acceptance toward food made from insects (36, 61).

#### Food Fussiness

Food fussiness, defined as the tendency to be a picky eater, was associated with a decreased acceptance to food products containing insect protein (55).

### Product Characteristics

For culturally unusual foods like insects, product characteristics considerably influence the acceptance among Western consumers (94). However, the studies reviewed not only examined the influence of certain attributes of insect-based products on consumer acceptance, but also the influence of consumers' perceived product characteristics of insect-based foods on their acceptance, which we have divided below into “perceived benefits” and “perceived risks.”

#### Preparer of Dish

According to the findings of a study of children from Denmark, whether the insect-based preparations are self-cooked or served does not make a difference in children's willingness to taste insect oatmeal balls (40).

#### Characteristics of Insect-Based Products

Various product characteristics such as price or taste were surveyed not only for general food choices (42, 45, 55, 62, 75, 87), but also specifically related to insect-based products.

The naturalness of a product has already been shown to be an important factor influencing willingness to consume other products (120, 161). However, naturalness was surveyed differently in the three studies that examined it (62, 70, 84). Ruby et al. (84) surveyed whether participants perceived the consumption of insects as natural for humans (e.g., “*It is not natural for humans to eat insects*.”) but could not show an effect on acceptance of insect-based products. In contrast, Lensvelt and Steenbekkers (70) surveyed whether respondents perceived insects as a natural product (e.g., “*I associate insects with natural food, without any additives or artificial ingredients*.”). According to Lensvelt and Steenbekkers (70), a positive association with the acceptance of insect-based foods has been revealed, which means the respondents perceived insects as a natural product. A study by Kornher et al. (62) examined the evaluation of food quality according to its naturalness, showing no effect on consumers' willingness to pay for a burger with grounded insects.

Price as a factor influencing the acceptance of insects as food has been investigated in eight studies (65, 69, 96, 104, 126, 144, 162). Elorinne et al. (126) and Kulma et al. (65) surveyed the influence of the price of insect-based foods compared to conventional products [e.g., “*I intend to use foods of insect origin if they are cheaper than meat*.” (126)], with both studies revealing a negative influence on acceptance. On the other hand, Pascucci and de-Magistris (162) and de-Magistris et al. (144) assessed the influence of four different prices (e.g., 1.50, 2.50, 3.50, 4.50 euro) on the willingness to pay for sushi with insects. Both studies demonstrated a negative impact of high prices on the willingness to pay for sushi with insects (144, 162). Tuccillo et al. (96) in turn surveyed whether the price would prevent consumers from including insects or insect-based products in their diet (e.g, “*Which of these reasons would make you desist from including insects and/or insect-based products in your diet?*”), showing a negative influence. In addition, Tuccillo et al. (96) were able to show that men were more likely to reject insect-based products due to a high price compared to women. Regarding an insect burger, Berger et al. (69) surveyed the influence of two price categories (low = 2.99 euro vs. high = 14.99 euro). Thereby, Berger et al. (69) considered different variables: expected quality, willingness to pay, and liking of the product. A positive influence of the price on the expected quality and the willingness to pay for an insect burger could be demonstrated. For the liking of the insect burger, the price was insignificant (69). In addition, Wilkinson et al. (104) also examined the influence of price. However, they did not investigate whether a high or low price has a negative or positive effect on acceptance, but whether three different consumer groups (“*neophobic consumers*,” “*neophilic consumers*,” and “*insect eating consumers”*) perceive price as an influencing factor. The results show that the consumer group “*insect eating consumers*” perceive the price as a more relevant influencing factor compared to the other two consumer groups (104).

#### Sensory Expectations and Ratings

An additional factor facilitating consumers' acceptance of insects as food is consumers' sensory expectations and ratings. Whereby two studies investigated general sensory expectations (77, 92), eight studies investigated the influence of aspects such as expected taste (41, 72, 80, 91, 96, 104, 153). In addition, Bartkowicz et al. (47) and Tan et al. (95) investigated the influence of sensory characteristics using specific products that participants were able to taste during the survey. For clarity, these studies are not presented in the description of the individual factors, instead they are described separately at the end of this section.

Palmieri et al. (77) and Sogari et al. (92) investigated the influence of general sensory expectations and both studies were able to show a positive influence on the acceptance of insect-based foods. More specifically, both studies surveyed how positively (or negatively) taste and appearance of insects-based food are evaluated and then formed an aggregated variable (77, 92).

Of the studies reviewed, four studies examined the influence of expected taste on the acceptance of insect-based foods, showing conflicting results (41, 96, 104, 153). For instance, a study by Cicatiello et al. (41) surveyed whether expected taste was perceived as a barrier to insect consumption (“*If you think about eating insect-based products, do you think that the following issues may be discouraging?*”), showing a negative influence: subjects perceiving taste as a barrier were less likely to consume insects. In contrast, Tuccillo et al. (96) assessed the extent to which taste would induce subjects to include insects or insect-based foods in their diets (“*Which of these reasons would make you include insects and/or insect-based products in your diet?*”) and could show a positive influence. Furthermore, Castro and Chambers (153) surveyed the impact of expected taste for participants from the United Kingdom, Spain, Australia, and the United States. A negative influence could only be shown for respondents from the United Kingdom, whereas the expected taste showed no influence for participants from Spain, Australia and the United States (153). The expected taste was also examined in the study by Wilkinson et al. (104). However, it was only investigated whether the expected taste has an influence (“*To what extent would the following factors influence your willingness to try eating insects?”*). Therefore, no statement can be made about the direction of the influence (104). In addition, Wilkinson et al. (104) compared the assessments of the influence of taste concerning to three consumer groups (“*neophobic consumers*,” “*neophilic consumers*,” and “*insect eating consumers*”). The results suggest that expected taste influences consumer acceptance, with the “*insect eating consumers*” group more likely to perceive taste as an influencing factor compared to the other two consumer groups (104). Mancini et al. (72) also examined expected taste as an influencing variable in their study but used the similarity of taste of products containing insect powder to familiar foods as a reference. It could be shown that participants who expected insects to taste like familiar foods were significantly more willing to try them (72).

Two studies in the review investigated the influence of expected texture on the acceptance of insect-based foods (41, 153). In this regard, Castro and Chambers (153) investigated whether expected texture [“*The texture (of insect-based products) would be bad*.”] would be a reason for respondents not to consume insect-based products. Whereas, Cicatiello et al. (41) investigated whether expected texture would be a barrier to the willingness to consume insect-based products. In the study by Cicatiello et al. (41), the expected texture was insignificant for the acceptance. The results of Castro and Chambers (153) need to be considered a bit more in detail: for participants from the United Kingdom, Spain, and Australia, the expected texture had no influence. However, for participants from the United States, it was shown that the expected texture had a negative influence (153).

In addition, the influence of the expected appearance on the acceptance of insect-based foods was also investigated (41, 104, 153). Castro and Chambers (153) examined this factor (more specifically, the color of an insect-based product) as a reason for not consuming insect-based foods. Cicatiello et al. (41), as also previously described, examined whether expected appearance was a barrier to the willingness to consume foods from insects (“*If you think about eating insect-based products, do you think that the following issues may be discouraging?*”). Again, Wilkinson et al. (104) investigated whether expected appearance was perceived as an influencing factor for acceptance of insect-based foods. In the study by Castro et al. (153), expected appearance showed no influence on the acceptance of insect-based foods, whereas Cicatiello et al. (41) revealed a negative impact. The results of Wilkinson et al. (104) suggest that perceived appearance has an influence, with perceived appearance being a more important influencing factor for the “*insect eating consumer*” group compared to the other two consumer groups.

Food quality also emerged as a potentially relevant factor influencing the acceptance of edible insects: four of the five studies examining this factor found a positive influence of food quality on the acceptance of insect-based products (70, 80, 91). However, food quality was surveyed differently in these four studies. Lensvelt and Steenbekkers (70) investigated the importance of quality of a food product in daily food choices, while Berger et al. (91) as well as Berger et al. (80) surveyed expected quality related to a specific insect-based product (“*Based on the presented information, which quality do you expect the nutrition bar (prepared with yellow mealworms) to have?*” (91) and “*On the basis of the information available, what quality do you expect from this truffle (prepared with yellow mealworms)?*' (80)]. In addition, Wilkinson et al. (104) investigated whether food quality was considered by participants to be a factor influencing the acceptance of insect-based foods. The results suggest that expected food quality shows an influence on acceptance, with the “*insect eating consumers*” group being more likely to see food quality as an influencing factor compared to the other two groups (104).

Bartkowicz and Babicz-Zielińska (47) investigated the influence of taste, texture, appearance, and odor on the acceptance of three different insect-based protein bars. One protein bar was prepared with ground grasshoppers, whereas the other two protein bars were prepared once with whole mealworms and once with ground yellow mealworms (47). The participants tasted the three different protein bars and rated the taste, texture, appearance, and smell for the respective product. A positive influence of the smell and the taste could be proven for all three protein bars (47). In contrast, the appearance and texture had no impact on the acceptance of the three different protein bars (47). In a study by Tan et al. (95), two tastiness variables were surveyed for three different mealworm products: a meatball and a milk drink based on yellow mealworms, and whole mealworms. On the one hand, the expected tastiness of the three mealworm products was asked before tasting by showing pictures of the respective products [“*How tasty do you expect it to be*?” (95)]. Second, respondents were then asked to taste the meatball and the milk drink and again rate the tastiness of the two products after sampling [“*How tasty do you experience it?*” (95)]. The influence of expected and experienced tastiness was investigated for both one-time and regular consumption. Both expected and experienced tastiness were found to have a positive influence on one-time and regular consumption (95).

#### Degree of Visibility of Insects and Amount of Insect Substitute

An important product characteristic of insect-based foods is the degree to which insects are visible in the product. In many studies (15.1%), it was found that respondents preferred insect-based products in which no insects or parts of them were visible compared to products in which insects or parts of them were visible (48, 52, 56, 59, 65, 70, 84, 86, 87, 92, 94, 96, 117, 144, 162–164). A single opposing result was reported by Modlinska et al. (149), whose participants favored products with easily discernible insect elements. In addition, Circus and Robison (43) were unable to show any influence of the degree of visibility of insects on the acceptance of insect-based products.

Not only the degree of visibility, but also the amount of insect substitute proved vital to the liking of an insect-based food product (39, 46, 102, 165). The studies mentioned above have all examined the proportion of insect substitutes in different ways. Thus, the results need to be considered in detail due to different survey methods. For example, both Biró et al. (102) and Castro et al. (39) studied house cricket flour as a substitute. However, Biró et al. (102) used cricket-enriched oat biscuits as the final product, while Castro et al. (39) chose chocolate chip cookies. Furthermore, Biró et al. (102) used four different amounts of house cricket flour in the biscuits (i.e., 0, 5, 10, and 15 g per 100 g flour blend). In contrast, Castro et al. (39) used three concentrations (i.e., 0, 43.2, and 86.4 g). In addition, Biró et al. (165) have studied pasta with silkworm flour as a substitute as the final product. The authors differentiated between three different amounts (i.e., 0, 5, and 10 g per 100 g of pasta). In turn, Delicato et al. (46) studied black soldier fly larvae fat at three different concentrations (i.e., 0, 25, and 50%) as insect substitutes. The authors investigated three end products: cakes, cookies, and wafers. Unanimously, the results of the studies by Biró et al. (102), Castro et al. (39), and Delicato et al. (46) indicate that acceptance is higher toward the tested products with lower amounts of insect substitutes. Only Biró et al. (165) showed in their study that the pasta with 10 g of insect substitute was preferred over those with 0 and 5 g.

Since the findings regarding the preferred amount of insect substitute vary across the literature, Ardoin et al. (166) conducted a sensory threshold study applying different rejection-type threshold methodologies to address this issue. According to their findings, the flavor of snack crackers changed noticeably when more than 5.8% whole-wheat flour was substituted with cricket powder. Thresholds for overall liking, texture, and color were 10.6, 15.6, and <20%, respectively (166). Furthermore, their results suggest that cricket-containing snack crackers would begin to reach a 25% rejection tolerance at approximately 15% whole-wheat flour substitution with cricket powder (i.e., 25% of their participants rejected the snack cracker with ~15% of the whole-wheat flour substituted) (166). Nevertheless, these insights cannot be generalized to all insects, as the insect species substantially influences consumers' acceptance (40, 50, 65, 84, 86, 109).

#### Insect Species and Life Stage

Whether consumers prefer specific insect species in foods was investigated in six studies (40, 50, 65, 84, 86, 109). While five studies were able to highlight an influence of insect species on the acceptance of insect-based products (40, 50, 84, 86, 109), three studies were unable to reveal any influence (40, 65, 109). Fischer and Steenbekkers (50) compared the willingness to eat 17 different insect species by presenting subjects with the names of the insect species without pictures. The 17 insect species included more broadly liked insects (e.g., grasshoppers and butterflies), more neutral or ambivalently regarded insects (e.g., crickets and moths), infrequently mentioned insects (e.g., termites and water bugs), and generally disliked insects (e.g., cockroaches and wasps). The results of Fischer and Steenbekkers (50) indicate that people in the Western world are more likely to accept crickets, grasshoppers, and mealworms, which may be due to their frequent marketing as food. While Chow et al. (40) surveyed the influence of insect species on the willingness to consume oatmeal balls with mealworm powder or grasshopper pieces, Schäufele et al. (86) compared differences in the willingness to try yellow mealworms and migratory locusts (*Schistocerca gregaria*). More in detail, they investigated the influence of insect species in three different forms of preparation: whole insects, crushed insects, and meatballs with insects (86). In a study by Chow et al. (40) no effect of insect type on the willingness to try the oatmeal balls was shown, but the taste of oatmeal balls with mealworm powder was rated more positively compared to those with grasshopper pieces. Schäufele et al. (86) were able to demonstrate for all three preparation forms that the willingness to try was higher for products with yellow mealworms than for those with migratory locusts. In addition, they revealed that the effect of insect type on willingness to try was greater for whole insects than for crushed insects or meatballs with grounded insects (86). Moreover, Ruby et al. (84) compared the willingness to consume seven different insect species, measuring the willingness to eat two forms of preparation (i.e., whole insects and cookies with insect flour). The following insect species were surveyed: ants, cockroaches, mealworms, crickets/grasshoppers, caterpillars, flies, and beetles. According to the results, for both preparation forms, the highest willingness to eat could be revealed for ants and the lowest for cockroaches (84). Moreover, Caparros Megido et al. (109) showed an influence of insect species on acceptance: when comparing cooked yellow mealworms with cooked house crickets, no difference in liking was found. In contrast, baked mealworms were preferred over baked house crickets (109). Kulma et al. (65) did not directly survey preference for specific insect species, but for five “*insect groups*”: [1] cockroaches, [2] beetles and bugs, [3] ants and termites, [4] beetle larvae, and [5] crickets, katydids and locusts. Participants were able to express their preference for consumption of the insect groups regardless of preparation on the one hand, and for grounded, unprocessed, and culinary processed preparation forms on the other hand (65). No influence of insect species could be demonstrated for the insect flour, but for the culinary processed dish it could be shown that crickets, katydids and locusts were preferred. For cockroaches, the lowest willingness to consume could be demonstrated (65).

The influence of different life stages of insects was investigated in only one study (96). When consuming whole insects (i.e., cricket, giant water bug, grasshopper, bee larva, mealworm, and silkworm), the results indicate that insects in an adult, live stage are preferred (i.e., crickets, giant water bugs, and grasshoppers). For insect-based products or dishes such as muffins or pasta, no effect of life stage on acceptance was demonstrated (96). However, because this has been investigated in only one study, further verification is needed.

#### Food Appropriateness

Rozin and Fallon (151) defined inappropriate foods as those that a cultural community considers unsuitable for consumption. Foods perceived as inappropriate are significantly less likely to be consumed. Consequently, the literature ascribes an important role to food appropriateness in the acceptance of insect consumption (94, 95, 117). All studies clearly showed that when insects are perceived as an appropriate food, their acceptance is also high (94, 95, 117).

#### Carrier Product Characteristics

A further possible method to foster familiarity, thereby reducing aversion toward consuming insects, is the combination with familiar and tasty products (74, 117). According to Tan et al. (117) savory foods, regardless of their origin (79), are best suited for the incorporation of insects.

#### Perceived Benefits

Insect-based products have several health and environmental benefits when compared to other protein sources (see Section Introduction). How individuals perceive these benefits and whether this has an impact on their acceptance of insect-based foods was investigated for the following three domains: health, nutrients, and the environment (43, 44, 53, 57, 70, 72, 73, 78, 83, 84, 86, 104, 108, 126, 140).

We found that perceived health benefits may be relevant for the acceptance of insects as an alternative protein source, as a positive effect was indicated by all three studies examining perceived health benefits (70, 72, 73).

The perceived nutritional benefits of insect consumption significantly increased acceptance in four out of six studies (78, 84, 104, 126). Conti et al. (44) and Higa et al. (57) reported that perceived nutritional benefits exerted no effect on acceptance of insects as food. All six studies explicitly analyzed the perception of the nutrient content of insect-based food in general or of a specific nutrient such as essential amino acids (44). For example, Ruby et al. (84) used the following item for assessing consumers' perception of nutrient content: “*Insects are highly nutritious*.”

A perceived low environmental impact associated with insects as food positively influenced consumers' acceptance of edible insects according to all studies focusing on the investigation of this factor (72, 73, 84). For example, Ruby et al. (84) surveyed the perceived environmental benefits of insects via the item “*Eating insects is good for the environment*.” whereas Mancini et al. (72) used the following item “*Eating products containing insect powder has positive effects on the environment*.” Menozzi et al. (73) surveyed their respondents' agreement with the positive effects on the environment. However, Ruby et al. (84) imposed a limitation on their finding, stating that the positive influence of a perceived low environmental impact on consumers' acceptance was only observed for whole, unprocessed insects. In the case of flour based on mealworms, no influence on acceptance could be revealed.

In addition to examining the perceived benefits divided according to the individual dimensions, these were also analyzed together as an aggregated variable. Six of the seven studies that examined this aggregated variable found that perceived benefits had a positive influence on the acceptance of insects as food (43, 53, 70, 83, 108, 140). However, the study by Schlup and Brunner (87) could not reveal any influence on the acceptance of insect-based products.

#### Perceived Risks

The concept of insects as a food product commonly evokes negative connotations such as dirty, unhygienic, and unhealthy (12). Accordingly, Westerners with limited knowledge on entomophagy may perceive the consumption of insects as risky and damaging to health.

Subjective risks commonly associated with the consumption of insects are allergic reactions, intoxication, and diseases transmitted by microbes (38). The influence of perceived risks seems to vary across the literature. According to seven out of ten studies examining subjective risks as an influence on the acceptance of insects as food, people convinced of the health-damaging consequences of insect consumption are significantly more reluctant to eat insects (76, 77, 84, 140, 145). Orsi et al. (76) reported that the influence of perceived risks was limited to processed insects. The findings of Ruby and Rozin (83) and Russell and Knott (85) are exceptions among the literature on this influence, as they determined that perceived risks were an irrelevant factor for the acceptance of insects as food.

Conversely, people with a higher tolerance to risks (i.e., preference for risky activities like bungee jumping) were found to have a greater probability of eating insect-based food products in the future (84).

In contrast, consumers' perceived self-infectability (the degree to which they consider themselves susceptible to infections) had no effect on their willingness to eat insects (84).

Perceived moral concerns related to the consumption of insects were investigated in four studies (82, 83, 85). Ruby and Rozin (83) and Russell and Knott (85) examined pain perception, cognitive ability, and immorality of killing in insects as perceived moral concerns [e.g., “*Insects can feel pain*.” (85)], while Rozin and Ruby (82) surveyed the immorality of killing insects (e.g., “*Killing this insect is immoral*.”). In the study by Ruby and Rozin (83) no influence of perceived moral concerns on the acceptance of insect-based foods was revealed, and Rozin and Ruby (82) showed a negative influence on acceptance. However, Rozin and Ruby (82) limited the measurement of the effect of perceived moral concerns to the thought of killing butterflies, suggesting that perceived moral concerns are insect species-specific. Russell and Knott (85) reported an effect of the dependent variable on the results: when they tested for willingness to substitute or pay, perceived moral concerns turned out to be insignificant, but when testing for willingness to eat, perceived moral concerns had a significant negative effect (85).

### Food Choice Motives

To understand the drivers of and barriers to the consumption of insect-based foodstuff in Western societies, it is important to assess the motivation behind consumers' food choices. This section includes our results related to the nine food choice motives (FCMs) according to Steptoe et al. (167): [1] familiarity, [2] sensory appeal, [3] ethical concerns, [4] natural content, [5] health, [6] convenience, [7] price, [8] weight control, and [9] mood. To these, we added a sustainability factor [10], since both Grasso et al. (55) and Onwezen et al. (75) applied a modified FCM questionnaire that included sustainability as an FCM. Furthermore, we included a factor called value for money [11], which measures the importance of receiving a good value for the money spent, following Schlup and Brunner (87), who complemented the list of FCMs with this factor. All FCMs are surveyed by assessing the importance of each motive in daily food choices by consumers using the following introductory sentence: “*It is important to me that the food I eat on a typical day…*” (167).

The FCM familiarity describes the importance of familiarity with the product in everyday food choices [e.g., “…*is what I usually eat*.” (167)]. Only Schlup and Brunner (87) examined the FCM familiarity and were able to demonstrate a negative influence on the acceptance of insect-based foods. Thus, it can be assumed that participants who scored high on the FCM familiarity are significantly less likely to include insects in their diet (87).

The FCM sensory appeal refers to the importance of taste, appearance, texture and smell in food choices [e.g., “…*smells nice*.” (167)]. Seven studies investigated the effect of the importance of sensory properties in daily food choices on the acceptance of insect-based foods by Western consumers (42, 45, 55, 62, 75, 87). Four studies explored sensory appeal in general (55, 75, 87). The results, however, are inconclusive. One study declared a positive influence (75), two studies reported no connection (75, 87), and one study argued that placing importance on sensory aspects in daily food choices hampers the acceptance of insects as food (55). In addition to studies that examined sensory appeal as an aggregate influencing variable, Cicatiello et al. (42) and Kornher et al. (62) examined the significance of specific aspects of sensory appeal. Although the original scale of Steptoe et al. (167) was not used to survey the sub-aspects of sensory appeal as in the other studies, quite similar survey methods were used by Cicatiello et al. (42) and Kornher et al. (62). While Kornher et al. (62) only surveyed the importance of taste related to food products in general, Cicatiello et al. (42) assessed the importance of taste, appearance, and texture related to food products in general. The results of both Cicatiello et al. (42) and Kornher et al. (62) indicate that the importance of taste in daily food choices does not influence the acceptance of insect-based foods. The importance of appearance in food choices was also insignificant regarding the acceptance of consuming insect (42). In contrast, the importance of texture in daily food choices was found to reduce acceptance of insect-derived products (42). Furthermore, a comparative study by De Boer et al. (45) investigated the influence of taste-oriented FCMs on the willingness to eat snacks from different alternative protein sources. Taste-oriented persons attribute high importance to taste and the meal itself (Sample item: “*She feels proud of her taste. She believes that her food choices are very attractive*.”), while reflection-oriented consumers consider on their meals as well as the ingredients [Sample item: “*She is very mindful of food. She wants to eat sensibly*.” (45)]. According to their results, consumers with taste-oriented FCMs have an increased probability of choosing a snack made from insects over a lentil- or hybrid meat-based snack (45).

The FCM natural content describes the importance for daily food choices that products contain natural ingredients [e.g., “…*contains no artificial ingredients*.” (167)]. A preference for natural contents, which was investigated in three studies, was found to be insignificant for the acceptance of edible insects (75, 87).

The results of two studies concerning the FCM ethical concern indicate that this FCM tends to be not significant for insect consumption (87, 108). Ethical concerns were collected using the three items “*Comes from a country I approve of politically*.” “*Has the country of origin clearly marked.”* and “*Is packaged in an environmentally friendly way*.” (168).

For the FCM convenience orientation examined by five studies (2, 55, 75, 87, 108), two of the studies demonstrated a positive (2, 87) and three demonstrated a non-significant influence (55, 75, 108). The FCM convenience orientation describes the importance in food choices that a portion of food can be easily prepared (e.g., “…*is easy to prepare*.”) and available everywhere (e.g., “…*is easily available in shops and supermarkets*.” (167)]. Note, however, that Verbeke (2) and Schlup and Brunner (87) collected convenience orientation using a different scale [e.g., “*At home, I preferably eat meals that can be prepared quickly*.” (169)], which is, however, very similar to the original scale of Steptoe et al. (167).

We found that health as an FCM may be relevant for the acceptance of insects as an alternative protein source, as a positive impact was indicated by three of the five studies examining healthiness (75, 79, 87), while the remaining two studies observed no connection to the acceptance of insects as food (55, 75). The FCM health describes the consideration of nutrient content and health benefits in daily food choices [e.g., “…*contains a lot of vitamins and minerals*.” (167)]. One variable that is closely associated with FCM health is the FCM weight control. This refers to the consideration of fat and calorie content and the possibility of weight control in the choice of food [e.g., “…*helps me control my weight*.” (167)]. According to the results of two studies in an article by Onwezen et al. (75) and the findings of Schlup and Brunner (87), it seems irrelevant whether consumers emphasize on weight control when choosing foods in the context of eating insects.

According to Onwezen et al. (75) consumers' mood when making food choices does not influence their probability of choosing insect-based foods, with mood describing whether food helps in coping with stress and improves mood [e.g., “…*helps me relax*.” (167)].

Three of the four studies that examined the FCM price found no influence of price as a motivation for daily food choices on the acceptance of insect-based products (41, 55, 75), while only one study revealed a positive effect on acceptance (75). Grasso et al. (55) and Onwezen et al. (75) surveyed the FCM price according to Steptoe et al. (167), raising the importance of a low price in daily food choices (e.g., “…*is not expensive*.”). In contrast, Cicatiello et al. (41) did not survey the original FCM price according to Steptoe et al. (167). Nevertheless, since the importance of price in daily food choices was also surveyed (e.g., participants were asked how much importance they attach to the price of foods in general), the results were assigned to the FCM price.

Only the study by Schlup et al. (87) examined the influence of the FCM value for money, showing no influence on the acceptance of insect-based foods. The FCM value for money describes the importance of cost/performance ratio in daily food choices [e.g., “*I compare prices between product variants in order to get the best value food*.” (170)].

Grasso et al. (55) and the three independent studies in Onwezen et al. (75) measured sustainability as an FCM. It describes how important the environmental friendliness of the product is in daily food choices (55, 75). Three of the four studies yielded results indicating that sustainability as an FCM did not affect the intention to consume food products containing insects (55, 75). Only one study, published in the article by Onwezen et al. (75), suggested a positive influence of sustainable food choices on the intention to consume insect-based food products. However, the studies used different survey methods: Grasso et al. (55) used three items to survey the FCM sustainability [e.g., “…*is environmentally friendly*.”), while Onwezen et al. (75) used only two items (e.g., “…*is produced in an environmentally friendly way*.”).

### Information

Providing information about the nutritional and environmental benefits of entomophagy is a common intervention aimed to increase Westerners' acceptance of edible insects (25).

#### Information Settings

Research suggests that providing information such as nutritional information, health claims, taste, sustainability issues, and ingredient information reduces the fear associated with trying insect-based food products (23, 25). Eight intervention studies in this review support the positive impact of providing information on entomophagy (36, 67, 72, 74, 88, 100, 112, 115), whereas three studies did not identify any influence of information (70, 162, 163). However, the type of information provided to the participants in the studies differed. Thus, Ardoin and Prinyawiwatkul (36) provided information on the safety, environmental friendliness, and nutrient content of insects compared to conventional meat. In contrast, in a study by Schouteten et al. (88) the participants were informed before the tasting session that the burger patty contained insects, while the other respondents were not informed. Through the results of the studies, it is not possible to draw general conclusions about whether health or environmental information has a greater influence on the acceptance of insect-based foods. Only three studies have investigated simultaneously the influence of different types of information: Lombardi et al. (115) and Verneau et al. (100) examined the influence of information on individual and societal benefits of consuming insects. Nevertheless, Verneau et al. (100) were unable to demonstrate a difference between the two types of information. However, in a study by Lombardi et al. (115), individual information had a greater influence than societal information. In contrast, Lensvelt and Steenbekkers (70) examined the influence of three different foci of information on acceptance toward insect-based foods. Respondents were given information on product-related factors, social norms, trust, or physiological factors, with a control group receiving no information. In doing so, Lensvelt and Steenbekkers (70) were unable to show any difference in their participants' preference for the product between the four different groups.

Additionally, Lensvelt and Steenbekkers (70) reported varying degrees of trust in information depending on the source of information. Their participants believed that information was trustworthy when provided by scientific researchers, close relatives, the government, and individuals who have previously consumed insect products, but not when it was provided by celebrities or food producers (70).

The literature seems to be divided over which is the most influential information strategy to reduce aversions to insects as food. Berger et al. (80) argued that disgust-based rejection of edible insects as food is best addressed with appeals to hedonic experience rather than with utilitarian reasons such as sustainability or long-term healthiness. Their statement is in line with Pascucci and de-Magistris (162), whose intensive use of positive frames associated with the social and environmental benefits of consuming insect-based foods did not significantly impact their participants' willingness to pay for insect-based food products. Moreover, Cavallo and Materia (164) reported drawback effects and a significant decrease in consumers' willingness to buy silkworm protein-based drinks when they received information on the high protein content of edible insects.

#### Package Design

The design of packaging is an integral part of marketing (171, 172). As such, several studies in our review have investigated the extent to which package design influences customers' purchase intentions (62, 144, 145, 149, 162, 164).

Three independent studies included in a paper by Baker et al. (145) explored the effect of explicit descriptions and images on product packaging in the retail setting, as well as the inclusion of equivalent information on restaurant menus. Based on their findings, Baker et al. (145) argued for the inclusion of images of processed (i.e., powdered) insects and the insect's scientific name (“*Nepomorpha”* instead of “*Giant Waterbug”*). Explicit descriptions in the form of the insect's common name and images of whole insects should be avoided since they increase perceived risks and significantly decrease the intention to consume insects (145).

Information about the insect content of a product in the form of a logo increased consumers' willingness to pay for edible insect products in two studies (144, 162). Both studies have used the logo called “*Chrysalide*,” which shows a stylized butterfly chrysalis (144, 162). In contrast, Modlinska et al. (149) showed an opposite result: a label that indicated the content of insects reduced the acceptance toward insect-based products.

According to Cavallo and Materia (164), a certificate on the product confirming its environmental friendliness does not increase consumers' willingness to buy insect-based products. In contrast, nutritional information on the packaging was found to positively influence consumers' willingness to pay for insect-based products (144, 162). Both de-Magistris and Pascucci (144) and Pascucci and de-Magistris (162) provided participants with details of Omega-3 fatty acids content as nutritional information. In contrast to these findings from the Netherlands, Kornher et al. (62) concluded in a German sample that the importance of nutritional information on food products was not a significant driver of the acceptance of insect-based food.

Research on the influence of using opaque packaging to reduce fear did not observe any effects (164).

## Discussion

Insights into consumer acceptance of insects as a source of alternative protein and its drivers are needed to steer Westerners toward a more sustainable protein consumption. The topics and trends identified in this review can inform strategies for advancing the future market share of edible insects, displacing more of the demand for meat from conventional livestock and thus reducing the harm that its production entails. However, as more than 115 influencing factors on the acceptance of insect-based foods were identified during the review, it is not possible to discuss each of them and draw conclusions. Therefore, we focused on selected factors and decided not to proceed in a belly- but rule-guided manner based on the following two criteria. Thus, we discuss [1] all factors that were investigated in more than 10% of the studies, and additionally, [2] all factors that were investigated in at least three studies if they all showed the same consistent results. Note that factors other than those discussed here also might have a significant influence on the acceptance of insect-based products, although they have only been investigated in one or two studies. However, due to limited data availability and space in this paper, we refrained from drawing conclusions for these variables.

### Sociodemographic Factors

Research on consumer acceptance of edible insects has found significant sociodemographic variation in rates of acceptance and identified common characteristics of potential entomophagists. Age, gender, and education level were the most studied sociodemographic variables.

In this review, the results on the influence of age are inconclusive. Although some studies have demonstrated a negative effect of age on the acceptance of insect-based foods (2, 64, 65, 67, 71, 76, 77, 87, 92, 93, 97, 99, 115), most studies with adult participants over 18 showed no effect (39, 41, 42, 48, 51, 55, 56, 58, 61, 62, 64, 66, 72–74, 86, 104–106, 108, 109, 117). Interestingly, one of the few studies on the acceptance of insect-based foods among children and adolescents found a reverse trend, in that older adolescents showed higher acceptance than children (49). Consequently, one could assume that there is an average age range, probably between 15 and 35, when people are most receptive to insect-based products. Whether this is really the case would have to be verified in further studies, which would also provide important information for target group-specific advertising and marketing campaigns.

Regarding the influence of gender, many studies showed that men were more likely to accept foods made from insects (2, 36, 41, 44, 51, 55, 58, 61, 62, 65–67, 69, 71, 76, 77, 83, 84, 86, 87, 92–94, 96, 97, 99–101, 104, 105, 112, 114, 116, 117). In addition, the results suggest that the degree of processing is crucial for the influence of gender. In studies comparing the gender-specific acceptance of processed and unprocessed insect-based food, an influence could only be demonstrated for the latter, for which men showed a higher acceptance (66, 76). In that women have a higher disgust sensitivity (143, 158), it can be assumed that they find whole insects more disgusting (173) and are therefore less willing to accept them as food. Conversely, the results suggest that the difference in disgust sensitivity between women and men becomes irrelevant for processed products. Another reason could be that men are more out for sensation seeking (174) and have a more adventurous taste than women (2). This suggests that both product selection and the degree of processing of insect-based foods could be tailored gender-specific in advertisements and marketing campaigns.

As with age, the results on the influence of educational level are also inconclusive. According to the studies examined, the level of education has no (2, 51, 56, 61, 65, 66, 71, 76, 86, 87, 105) or a positive influence (41, 55, 62, 93, 99, 101, 107, 108) on the acceptance of insect-based foods. Furthermore, it is difficult to draw general conclusions and inferences for Western societies, as a direct comparison between countries is not always possible due to differences in school and education systems. The influence of education on the acceptance of insect-based foods was most frequently studied in Germany. Here, the trends on the influence of educational level are much clearer: four out of five studies were unable to demonstrate any influence (56, 66, 76, 86), while one study was able to reveal a positive effect (62). These findings show that the level of education should be considered on a country-specific basis rather than generalized across all Western societies. However, due to the limited data available, further research is needed in this area. In particular, there is a lack of studies examining the impact of formal educational programs in schools on increasing the acceptance of insect-based foods. Teaching materials on entomophagy that could be used for this purpose—and for use in universities—are already available (175–178).

Only the results on income clearly indicate no influence on the acceptance of insect-based foods, as all studies failed to demonstrate any impact (67, 87, 93, 105). However, note that income as a predictor has rarely been studied compared to other sociodemographic factors. Even if income itself has no influence, the results of the studies examined suggest that a high price is a barrier to the acceptance of insect-based products (65, 96, 126, 144, 162). In addition, one of the most important FCMs in many Western societies seems to be “*price*” (179). More specifically, “*price*” was the most important FCM in Spain, Greece, the Netherlands, Ireland and Portugal, and the second most important FCM in Germany, Poland and the United Kingdom, respectively (179). Income could thus possibly have an indirect influence: people with lower incomes will probably pay more attention to lower prices when buying insect-based foods (180).

### Personality and Emotional Factors

Personal and emotional influences proved to add considerable predictive power for the acceptance of insect-based foods (2, 49, 66, 75). The most frequently analyzed variables in these categories were familiarity, attitudes food neophobia, and insect eating disgust.

Higher familiarity with eating insects led to higher acceptance of insect-based foods in many studies (2, 37, 49, 51, 61, 67, 95, 100, 104–106, 112, 141). A simple but important implication is that to increase the acceptance of insects as food, familiarity must be increased. Approaches from quite different areas could be used to increase familiarity, for example through regional and national information and education campaigns, tasting events in city centers, street food festivals, and trade fairs, or by increasing the availability, visibility, and variety of insect-based foods in supermarkets, restaurants, and online stores. Integrating edible insects into popular dishes and common carrier products, while ensuring that the visibility of the insect is low, may also promote the consumption of insects in Western food markets (52, 70, 90, 113). With little or no insect-based foods on the market, and therefore missed opportunities for positive taste experiences, it will likely be difficult to increase familiarity and acceptance toward them. This factor seems even more important, as La Barbera et al. (127) suggested that increased familiarity also might reduce the negative effect of food neophobia. However, the results on familiarity must be viewed in a differentiated manner, as many different methods were used to survey this construct. Thus, interpretation and comparison of the results remain challenging (see Section Familiarity).

Attitudes toward eating insects have been shown to be a positive influencing factor in many studies (37, 49, 50, 70, 73, 75, 99, 100, 104, 107, 115, 126, 127). Furthermore, results show that attitudes differ between various insect products (139). For example, attitudes toward whole insects (e.g., freeze-dried buffalo worms) were less favorable compared to processed insect products (e.g., ground buffalo worms in burger patties) (139). The same trend could be shown for the taste expectations: compared to processed insect products, lower sensory and emotional expectations were associated with whole insects (52, 117). These results illustrate that the visibility of insects in food is a very important product characteristic that not only influences various other factors, but also directly affects the acceptance of insect-based foods (48, 52, 56, 59, 65, 70, 84, 86, 87, 92, 94, 96, 117, 144, 162–164). In some studies, attitudes were assessed using semantic differentials, and participants were found to have partly ambivalent attitudes, some of which mutually contradictory (49, 56). Thus, Dupont and Fiebelkorn (49) were able to show among German children and adolescents that an insect burger was rated as rather disgusting, but at the same time, however, it was perceived as environmentally friendly and healthy. In a study by Hartmann et al. (56) adult participants from Germany rated fried crickets and silkworms as primitive yet simultaneously nutrient-rich. In addition, the results of Fischer and Steenbekkers (50) showed that only affective attitudes had a positive influence on the acceptance of insect-based food, whereas cognitive and overall attitudes were not significant.

Overall, attitudes are crucial for the acceptance of insect-based foods. However, attitudes can vary greatly between different insect species and developmental stages, as well as from product to product. Even for the same product, the expression of a person's attitudes can sometimes be contradictory. Therefore, in addition to promoting positive attitudes toward insect-based foods in general, interventions should also focus on specific edible insects and products. In particular, focus should be placed on the affective dimension of attitudes with negative connotations, such as the perception of insect-based foods as disgusting, unhygienic, and dirty.

The negative influence of food neophobia has been shown not only for the acceptance of insect-based food (2, 40, 42, 49, 51, 56, 59, 61, 62, 66, 67, 71, 72, 74, 76, 84–87, 89, 92, 94–96, 99, 104, 108, 115–117, 127, 143), but also for many other novel foods such as cultured meat (25, 49, 120). To reduce food neophobia, exposure to novel foods should be provided (90, 181). Furthermore, taste training (182) and sensory education (183) may also reduce food neophobia.

Insects are not a traditional food in Western societies (109, 184, 185) and are often associated with disgust in these societies (186). Many studies have consistently shown that disgust with eating insects has a negative impact on the acceptance of insect-based foods (50, 52, 57, 72, 75, 76, 83–85, 103, 125, 127, 131, 153). To conclude that disgust with insects and insect-based foods must be reduced for them to succeed in the Western food market is straightforward. Implementation will not be so easy, however, because disgust with insects is a deeply embedded core emotion in Western societies (127) and shaped by culture, social norms and previous experiences (187). From our point of view, two paths can be taken: first, products and packaging should be designed in such a way that the consumer does not experience a disgust reaction. Products containing processed insects are expected to induce lower disgust reactions because their insect origin and disgusting attributes such as long legs with spines or a slimy body texture are less prominent (56). Explicit descriptions in the form of colloquial insect names and images of whole insects should also be avoided on the packaging and in advertising (145, 149); logos referring to the insect content could be used instead (144, 162). Second, one could try to directly reduce consumer disgust with eating insects, but that is probably more difficult to accomplish than the indirect route of avoiding disgusting products, packaging, and advertising. Educational measures in schools may contribute to familiarizing children with the concept of entomophagy and thus counteract the development of disgust toward the consumption of insects from an early age. This seems to be particularly effective because disgust is formed in childhood and hardly changes in adulthood (158, 188).

### Diet

All studies that have investigated the prior consumption of insects have been able to show that it increases the acceptance of insect-based foods (36, 37, 50, 59, 61, 64, 66, 70, 73, 77, 78, 86, 87, 92, 94, 99, 104–106, 108, 109, 112, 113, 117, 143, 144). Furthermore, the consumption of insects reduces disgust toward insects (113). Conversely, however, poor taste experiences can also lead to decreased acceptance of insect-based products, for example, by increasing disgust reactions (127, 189). This is where the snake bites its own tail, as individuals with high disgust levels are unlikely to try insect-based foods. However, repeated consumption of insect-based products with positive taste experiences is useful in solidifying positive attitudes and familiarity (106). In summary, we believe it is essential that consumers' first taste experiences with insect-based foods are positive. If the initial taste experiences are negative and further reinforce the aversion to insects that probably already exists, the likelihood that these consumers will ever try insect-based foods again is very low.

### Limitations

When critically assessing our review, two main limitations can be identified. A common limitation of a systematic review concerns the comparability across studies. The individual studies within our review all focused on consumer acceptance of edible insects. However, their research priorities differed, resulting in a plethora of different research methods and designs, with different sample compositions in terms of representativeness and specific target groups and various dependent variables. In the scope of our systematic review, these individual study characteristics could not be considered. Hence, a meta-analysis could be performed in the future to further synthesize this field of research. Furthermore, it would be helpful, if researchers in the field find ways to increase comparability across studies, for instance via the application of standardized measures or common theoretical frameworks. La Barbera et al. (131) already proposed such a standardized measurement for assessing attitudes, called the entomophagy attitude questionnaire.

A further limitation of this review is the remarkable variety of factors that may influence the acceptance of insects as food in Western countries: we identified a total 115 different factors throughout the body of literature (cf. Supplementary Table 1). Due to a missing common framework, we spotted several variables that had the same name, but measured different constructs. Likewise, we identified items measuring the same construct under different names. This limitation becomes clearly apparent when considering the variables knowledge, perceived benefits, and familiarity. In most studies, the item on familiarity with entomophagy was dichotomously coded with a statement asking whether participants had heard of the term entomophagy (2, 51, 66, 76). However, there are numerous measurements that blur the line between familiarity, knowledge, and perceived benefits. For instance, Kane and Dermiki (61) defined knowledge as having heard of the term entomophagy, whereas the description of knowledge by Woolf et al. (105) involves having “*heard about the benefits of entomophagy”* (p. 102). In other studies, this reasoning underlies the definition of familiarity and perceived benefits. The overlap and complexity of these three constructs illustrates the lack of accuracy of certain factors. In this review, we assigned these borderline cases to the variable that best matched their content, based on definitions in textbooks, dictionaries, and the papers that described and surveyed the constructs. However, future research would benefit from a common framework and unambiguous definitions of the constructs under study.

## Conclusion

The findings of this systematic review provide a comprehensive summary of the acceptance of edible insects in Western societies. The results reveal a complex picture of many different and related key factors influencing consumers' perceptions and the acceptance of insects as food. Nevertheless, the increasing interest in research on entomophagy as well as the very recent development of the European Food Safety Authority granting approval of the yellow mealworm and the migratory locust as a novel food support edible insects as a sustainable solution to the protein demands of a rapidly growing world population.

Research on consumer acceptance of insects as food has found demographic variation in rates of acceptance and identified several common objections and perceived benefits. Since this review article provides a comprehensive overview of the literature, the findings also revealed research gaps and lines for future research. Due to the wide range of variables tested in the body of literature and uncertainties regarding the influence of several variables, there remain questions to be answered by future research. Upcoming studies can expand our knowledge on the acceptance of insect-based foodstuff in Western societies by further elaborating on variables with controversial results or by studying factors that were previously only marginally examined. The table (cf. Supplementary Table 1) attached to this review provides an accurate summary of these variables.

Moreover, there is a need for future research with a comparative approach. Firstly, the comparison between multiple novel protein sources can help to specify target groups for different alternative protein sources. Secondly, comparisons between various products made from a specific alternative protein could promote our understanding of appropriate product combinations for a Western food market. Acknowledging the cultural diversity within the Western world, a third focus of future research should be cross-country comparisons.

Furthermore, a prominent research gap that sets the agenda for future research is the substantial disparity among quantitative studies, particularly in experimental study designs. Notably, the number of experimental studies was relatively small compared to the total number of studies on consumer acceptance. Conducting experiments in authentic real-life settings to evaluate the behavioral choices of potential consumers could be a further contribution to the research field.

Finally, it should be investigated whether and how the acceptance of insects as food and the different influencing factors have changed in recent years. Initial long-term studies of other novel foods, such as cultured meat, are already available (190). However, a corresponding study for the acceptance of insect-based foods is still pending.

## Data Availability Statement

The original contributions presented in the study are included in the Supplementary Material, further inquiries can be directed to the corresponding author.

## Author Contributions

TK: writing—original draft, formal analysis, data curation, visualization, and methodology. JD: writing—review & editing, conceptualization, methodology, and validation. LB: formal analysis, data curation, and validation. FF: writing—review & editing, conceptualization, methodology, resources, supervision, and project administration.

## Conflict of Interest

The authors declare that the research was conducted in the absence of any commercial or financial relationships that could be construed as a potential conflict of interest.

## Publisher's Note

All claims expressed in this article are solely those of the authors and do not necessarily represent those of their affiliated organizations, or those of the publisher, the editors and the reviewers. Any product that may be evaluated in this article, or claim that may be made by its manufacturer, is not guaranteed or endorsed by the publisher.
