# Supplementary material for: Acceptance of Insect-Based Food Products in Western Societies: A Systematic Review
Source: Front Nutr. 2022 Feb 21;8:759885. doi: 10.3389/fnut.2021.759885 (PMC8901202; doi:10.3389/fnut.2021.759885)
Supplement: Supplementary file 2 [file Data_Sheet_1.pdf]

Bibliography Mixed-Methods Approaches

Burt, K. G., Kotao, T., Lopez, I., Koeppel, J., Goldstein, A., Samuel, L., & Stopler, M. (2020). Acceptance of using cricket flour as a low carbohydrate, high protein, sustainable substitute for all-purpose flour in muffins. *Journal of Culinary Science and Technology*, *18*(3), 201–213. https://doi.org/10.1080/15428052.2018.1563934

Circus, V. E., & Robison, R. (2019). Exploring perceptions of sustainable proteins and meat attachment. *British Food Journal*, *121*(2), 533–545. https://doi.org/10.1108/BFJ-01-2018-0025

Fasanelli, R., Galli, I., Riverso, R., & Piscitelli, A. (2020). Social representations of insects as food: An explorative-comparative study among millennials and x-generation consumers. *Insects*, *11*(10), 656.

Jones, V. (2020). "Just don't tell them what's in it": Ethics, edible insects and sustainable food choice in schools. *British Educational Research Journal*, *46*(4), 894–908. https://doi.org/10.1002/berj.3655

Kane, B., & Dermiki, M. (2021). Factors and conditions influencing the willingness of Irish consumers to try insects: a pilot study. *Irish Journal of Agricultural and Food Research*, 1–16. https://doi.org/10.15212/ijafr-2020-0126

Kostecka, J., Konieczna, K., & Cunha, L. M. (2017). Evaluation of insect-based food acceptance by representatives of polish consumers in the context of natural resources processing retardation. *Journal of Ecological Engineering*, *18*(2), 166–174. https://doi.org/10.12911/22998993/68301

Nyberg, M., Olsson, V., & Wendin, K. (2020). Reasons for eating insects? Responses and reflections among Swedish consumers. *International Journal of Gastronomy and Food Science*, *22*(October), 100268. https://doi.org/10.1016/j.ijgfs.2020.100268

Ritger, S., Mirosa, M., Mangan-Walker, E., & Clarkson, C. (2016). Entomophagy: Understanding New Zealand consumers' attitudes toward eating insects. *Locale - The Australasian-Pacific Journal of Regional Food Studies*, *6*, 20–40.

Rozin, P., & Ruby, M. B. (2020). Bugs are blech, butterflies are beautiful, but both are bad to bite: Admired animals are disgusting to eat but are themselves neither disgusting nor contaminating. *Emotion*, *20*(5), 854–865. https://doi.org/10.1037/emo0000587
