# Supplementary material for: Acceptance of Insect-Based Food Products in Western Societies: A Systematic Review
Source: Front Nutr. 2022 Feb 21;8:759885. doi: 10.3389/fnut.2021.759885 (PMC8901202; doi:10.3389/fnut.2021.759885)
Supplement: Supplementary file 3 [file Data_Sheet_2.pdf]

Bibliography Qualitative Papers

Balzan, S., Fasolato, L., Maniero, S., & Novelli, E. (2016). Edible insects and young adults in a north-east Italian city an exploratory study. *British Food Journal*, *118*(2), 318–326. https://doi.org/10.1108/BFJ-04-2015-0156

Bogueva, D., & Marinova, D. (2020). Cultured meat and australia's generation Z. *Frontiers in Nutrition*, *7*(September), 1–15. https://doi.org/10.3389/fnut.2020.00148

Brynning, G., Bækgaard, J. U., & Heckmann, L. H. L. (2020). Investigation of consumer acceptance of foods containing insects and development of non-snack insect-based foods. *Industrial Biotechnology*, *16*(1), 26–32. https://doi.org/10.1089/ind.2019.0028

Clarkson, C., Mirosa, M., & Birch, J. (2018). Consumer acceptance of insects and ideal product attributes. *British Food Journal*, *120*(12), 2898–2911. https://doi.org/10.1108/BFJ-11-2017-0645

Gallen, C., Pantin-Sohier, G., & Peyrat-Guillard, D. (2019). Cognitive acceptance mechanisms of discontinuous food innovations: The case of insects in France. *Recherche et Applications En Marketing*, *34*(1), 48–73. https://doi.org/10.1177/2051570718791785

House, J. (2016). Consumer acceptance of insect-based foods in the Netherlands: Academic and commercial implications. *Appetite*, *107*, 47–58. https://doi.org/10.1016/j.appet.2016.07.023

House, J. (2019). Modes of eating and phased routinisation: Insect-based food practices in the netherlands. *Sociology*, *53*(3), 451–467. https://doi.org/10.1177/0038038518797498

Jones, V., & Beynon, S. (2020). Edible insects: applying Bakhtin's carnivalesque to understand how education practices can help transform young people's eating habits. *Children's Geographies*, 1–11. https://doi.org/10.1080/14733285.2020.1718608

Lupton, D., & Turner, B. (2018). Food of the future? Consumer responses to the idea of 3D-printed meat and insect-based foods. *Food and Foodways*, *26*(4), 269–289. https://doi.org/10.1080/07409710.2018.1531213

Myers, G., & Pettigrew, S. (2018). A qualitative exploration of the factors underlying seniors' receptiveness to entomophagy. *Food Research International*, *103*, 163–169. https://doi.org/10.1016/j.foodres.2017.10.032

Nyberg, M., Olsson, V., & Wendin, K. (2020). 'Would you like to eat an insect?'-Children's perceptions of and thoughts about eating insects. *International Journal of Consumer Studies*. https://doi.org/10.1111/ijcs.12616

Sahakian, M., Godin, L., & Courtin, I. (2020). Promoting "pro", "low", and "no" meat consumption in Switzerland: The role of emotions in practices. *Appetite*, *150*. https://doi.org/https://doi.org/10.1016/j.appet.2020.104637

Simion, V. E., Dourado Martins, O. M., Tudor, L., Mitranescu, E., & Zamfirache, I. (2020). Consumption of edible insects - factors influencing individuals to try new foods. *Revista Romana De Medicina Veterinara*, *30*(3), 44–50.

Sogari, G., Menozzi, D., & Mora, C. (2017). Exploring young foodies′ knowledge and attitude regarding entomophagy: A qualitative study in Italy. *International Journal of Gastronomy and Food Science*, *7*, 16–19. https://doi.org/10.1016/j.ijgfs.2016.12.002

Sogari, Giovanni. (2015). Entomophagy and Italian consumers: An exploratory analysis. *Progress in Nutrition*, *17*(4), 311–316.

Sogari, Giovanni, Bogueva, D., & Marinova, D. (2019). Australian consumers' response to insects as food. *Agriculture*, *9*(5). https://doi.org/10.3390/agriculture9050108

Tan, H. S. G., Fischer, A. R. H., Tinchan, P., Stieger, M., Steenbekkers, L. P. A., & van Trijp, H. C. M. (2015). Insects as food: Exploring cultural exposure and individual experience as determinants of acceptance. *Food Quality and Preference*, *42*, 78–89. https://doi.org/10.1016/j.foodqual.2015.01.013

Tucker, C. A. (2014). The significance of sensory appeal for reduced meat consumption. *Appetite*, *81*, 168–179. https://doi.org/10.1016/j.appet.2014.06.022
