# Supplementary material for: Acceptance of Insect-Based Food Products in Western Societies: A Systematic Review
Source: Front Nutr. 2022 Feb 21;8:759885. doi: 10.3389/fnut.2021.759885 (PMC8901202; doi:10.3389/fnut.2021.759885)
Supplement: Supplementary file 4 [file Data_Sheet_3.pdf]

Acceptance of Insect-Based Foodstuff in Western Societies: A Systematic Review and Comprehensive Update

Search string Scopus:
(TITLE-ABS-KEY(accept* OR willing* OR consum* OR intention)) AND (TITLE-ABS-KEY("edible insects" OR "edible insect" OR "entomophagy" OR "consume insects" OR "eating insects" OR "insect-based" OR "insects as food" OR "insect food")) AND (LIMIT-TO (PUBSTAGE,"final")) AND (LIMIT-TO (DOCTYPE,"ar")) AND (LIMIT-TO (LANGUAGE,"English"))
Results: 603

Search string Web of Science:
(TS=(accept* OR willing* OR consum* OR intention)) AND (TS=("edible insects" OR "edible insect" OR "entomophagy" OR "consume insects" OR "eating insects" OR "insect-based" OR "insects as food" OR "insect food"))
Restrict: English, Article
Results: 579

Search string Science Direct:
(acceptance OR willingness OR consumption) AND ("edible insects" OR "entomophagy" OR "consume insects" OR "eating insects" OR "insect-based" OR "insect food")
Restrict: Research Article
Results: 862

Search string JSTOR:
((("edible insect*" OR "entomophagy" OR "consume insects" OR "eating insects" OR "insect-based" OR "insects as food" OR "insect food")) AND ((accept* OR willing* OR consum* OR intention))) AND la:(eng OR en)
Restrict: Article, Journal
Results: 2.122
Imported: 1000

Search string Google Scholar:
(acceptance OR willingness OR consumption OR intention) AND ("edible insects" OR "entomophagy" OR "consume insects" OR "eating insects" OR "insect-based" OR "insect food")
Restrict: English
Results: 16.600
Imported: 1000
